# Integrative Omics Defines Metabolic Biomarkers and Genetic Regulatory Mechanisms of Mortality Risk

**DOI:** 10.1002/advs.202514464

**Published:** 2025-11-18

**Authors:** Peihao Liu, Bingxing An, Jumei Zheng, Qiao Wang, Zhirui Yang, Zhengda Li, Dawei Liu, Fan Ying, Jie Wen, Lingzhao Fang, Guiping Zhao

**Affiliations:** ^1^ Institute of Animal Sciences Chinese Academy of Agricultural Sciences Beijing 100193 China; ^2^ Center for Quantitative Genetics and Genomics (QGG) Aarhus University Aarhus 8000 Denmark; ^3^ Mile Xinguang Agricultural and Animal Industrials Corporation MiLe 652300 China

**Keywords:** biomarkers, metabolomics, mGWAS, mortality risk

## Abstract

The genetic and metabolic architecture of mortality risk represents a fundamental, yet poorly understood, challenge in human medicine and livestock breeding. Here serum metabolomics and multi‐omics data is integrated in a designed 3‐generation chicken model (n = 1,277) with divergent mortality. The analysis reveals a trade‐off between heightened inflammatory responses and impaired growth in susceptible animals. To uncover the genetic underpinnings, 45,585 metabolite quantitative trait loci are identified, which are predominantly enriched among liver‐specific regulatory variants. Using a machine learning approach, a robust 16‐metabolite signature is established, including hexyl glucoside and pyrraline, that accurately predicts mortality risk. Importantly, these metabolites and their genetic loci offer practical targets for genomic selection in chicken breeding, providing a direct approach to enhance disease resistance and survival. Cross‐species comparison with human data revealed conserved metabolic dysregulation pathways, while also highlighting species‐specific immuno‐metabolic pathophysiology. Furthermore, the findings pinpoint butyrate‐mediated microbiota‐host interactions and the dual antioxidant functions of L‐cysteine as critical regulatory mechanisms. Together, these results delineate an evolutionarily conserved immuno‐metabolic framework for mortality risk, offering novel biomarkers for selective breeding and potential therapeutic targets for human metabolic diseases.

## Graphical abstract

Understanding mortality mechanisms remains a fundamental challenge. Through multi‐omics analysis of a three‐generation chicken model, are identified 45,585 mQTLs and establish a 16‐metabolite predictor of mortality. An inflammation‐growth trade‐off and evolutionarily conserved pathways involving butyrate–microbiota interactions and L‐cysteine's dual antioxidant roles are revealed, providing targets for genomic selection to breeding disease‐resistant poultry.

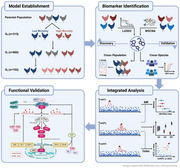

## 1 Introduction

Mortality risk represents a fundamental biomedical challenge across species, reflecting complex systemic failures that arise from an interplay of metabolic, immune, and genetic defects.^[^ ^1^ ^]^ This challenge is starkly illustrated in the global poultry industry, where high broiler mortality compromises economic viability and animal welfare amidst growing consumer demand.^[^ ^2^ ^]^ While environmental stressors (such as heat stress, disease, or overcrowding) are contributing factors, a central issue is an unintended consequence of intensive selection for rapid growth. Modern breeding has achieved a four‐fold increase in body weight, but at the cost of imposing a high metabolic burden that disrupts the body's internal homeostasis.^[^ ^3^, ^4^, ^5^ ^]^ This positions the chicken as an ideal model organism, where confounders like environment, diet, and even causes of death can be tightly controlled, enabling dissection of the genetic and metabolic architecture underlying mortality and its inherent trade‐offs.

Indeed, mortality is a polygenic trait influenced by a complex interplay of genetic, metabolic, environmental, physiological, and host‐microbiota factors.^[^ ^6^, ^7^ ^]^ Genome‐wide association studies have identified numerous loci associated with lifespan and age‐related mortality, including genes regulating metabolic processes, immune responses, and cardiovascular functions.^[^ ^8^, ^9^ ^]^ However, the causal genes/variants and molecular mechanisms (e.g., metabolic pathways through which these genomic variants act) underlying mortality are largely unknown. This is primarily due to the highly heterogeneous nature of mortality itself, which serves as a composite endpoint that masks diverse underlying causes such as specific diseases, physiological decline, or management‐related stressors, thereby obscuring the precise genetic mechanisms. Furthermore, this uncertainty is compounded by the limited explanatory power of individual loci, each typically accounting for less than 1% of phenotypic variance, and the inadequate functional annotation of associated genomic regions.^[^ ^10^, ^11^, ^12^ ^]^ The integration of multi‐omics approaches, such as metabolomic profiling and molecular QTL mapping, offers a promising framework for bridging this gap. These strategies enable the identification of biomarkers and the characterization of metabolic‐immune trade‐offs, thereby providing a functional context for genetic associations and enhancing our understanding of the molecular architecture underlying mortality.^[^ ^13^ ^]^

Therefore, to overcome these limitations, we conducted comprehensive serum metabolomic profiling using a well‐designed broiler model (n = 1,277) with a pedigree designed to segregate for mortality risk. Systematic differences emerged in mortality distribution, cytokine patterns, and metabolite profiles, which were subsequently confirmed through a replication set. Through metabolite genome‐wide association studies (mGWASs), we identified 45,585 metabolite quantitative trait loci and outlined a multi‐tissue regulatory framework underlying metabolic divergence by integrating molecular QTLs (molQTLs) from the Chicken Genotype‐Tissue Expression Project (GTEx).^[^ ^14^, ^15^ ^]^ These loci provide valuable candidate marker resources for breeding to enhance disease resistance in chickens. By employing machine learning, weighted correlation network analysis (WGCNA), and functional experiments, we discovered 16 prognostic biomarkers, which can be directly applied to individual selection strategies to improve flock survival and overall health status. Cross‐species analysis further connected these biomarkers to human complex diseases, specifically highlighting their evolutionarily conserved metabolic pathways: *PTER*‐dependent cysteine homeostasis regulation and butyrate‐mediated microbiota‐host crosstalk, demonstrating that mortality risk arises from metabolic‐immune system trade‐offs. This research has generated a comprehensive omics dataset, facilitating the elucidation of mechanisms underlying chicken mortality, supporting the development of precision breeding strategies, and informing research on human metabolic diseases.

## 2 Result

### 2.1 A Designed Selection Model Establishes Cohorts with Divergent Mortality Risk

**Figure 1:**
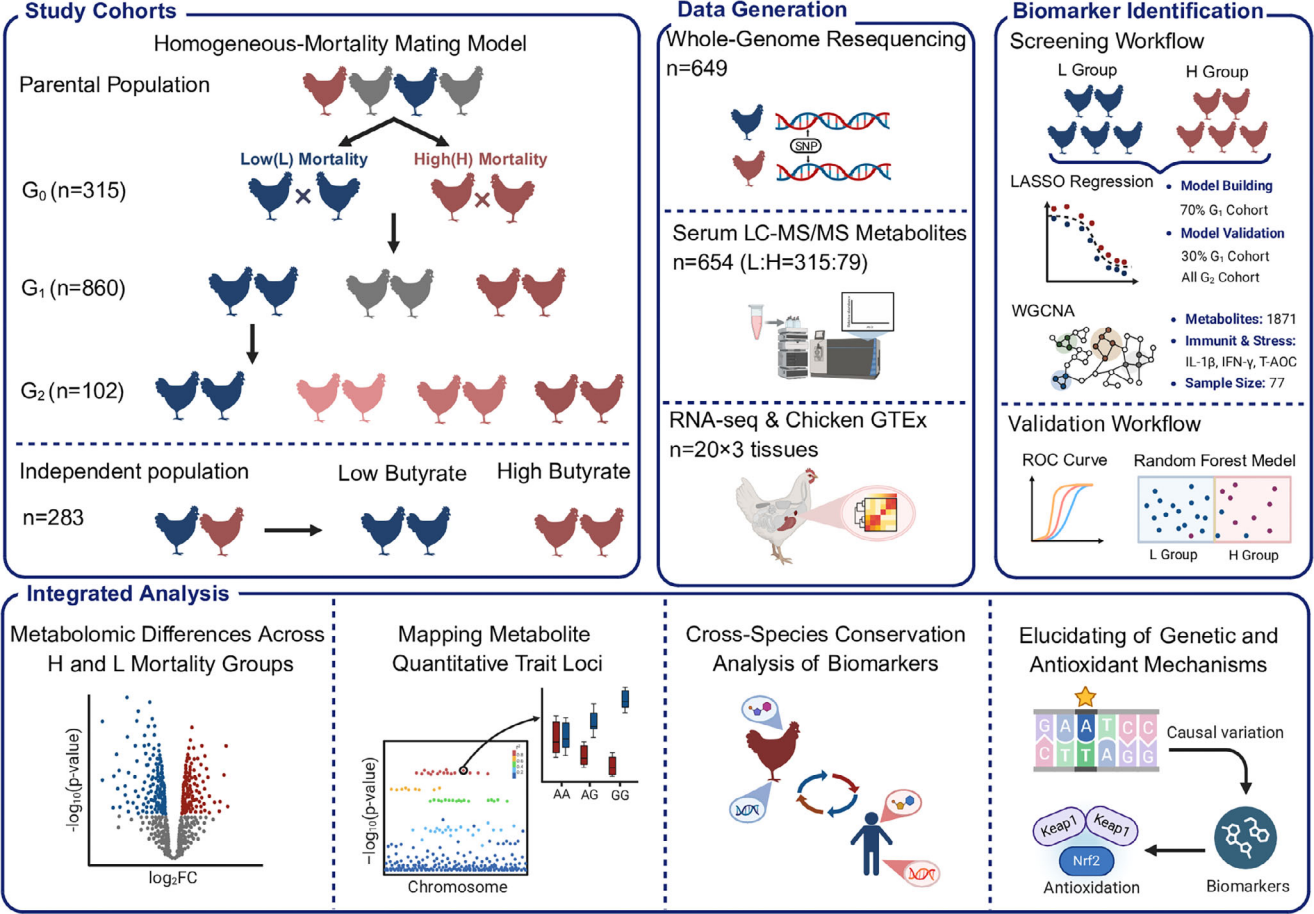
Schematic overview of the multi‐omics workflow for mortality risk biomarker discovery and validation. A multi‐generational chicken model (G_0_‐G_2_) was established for this study. In Cohort 1 (G_1_; n = 860 broilers from 181 full‐sib families), birds were classified into high‐mortality (H, n = 115) and low‐mortality (L, n = 406) groups. Comparative metabolomic profiling identified differential metabolites associated with mortality. Genome‐wide association studies were used to map metabolite quantitative trait loci for these metabolites. Least absolute shrinkage and selection operator (LASSO) regression and weighted correlation network analysis (WGCNA) were integrated to identify metabolic biomarkers linked to mortality risk. Biomarker‐based predictive models were trained in Cohort 1 and internally validated in Cohort 2 (G_2_, n = 102). Cross‐species validation was further conducted in an independent human extreme‐longevity cohort (Cohort 3), which included two sub‐cohorts: Verify 1 (n = 119) and Verify 2 (n = 37). Existing human evidence from probabilistic transcriptome‐wide association studies (PTWAS) and Mendelian randomization (MR) analyses confirmed evolutionarily conserved genetic regulation and physiological functions of these biomarkers. Mechanistic integration of microbiome data revealed butyrate‐mediated microbiota‐host crosstalk, and in vitro experiments elucidated the dual antioxidant mechanisms of L‐cysteine as a functional biomarker. Image created with BioRender.com and published with permission.

**Figure 2:**
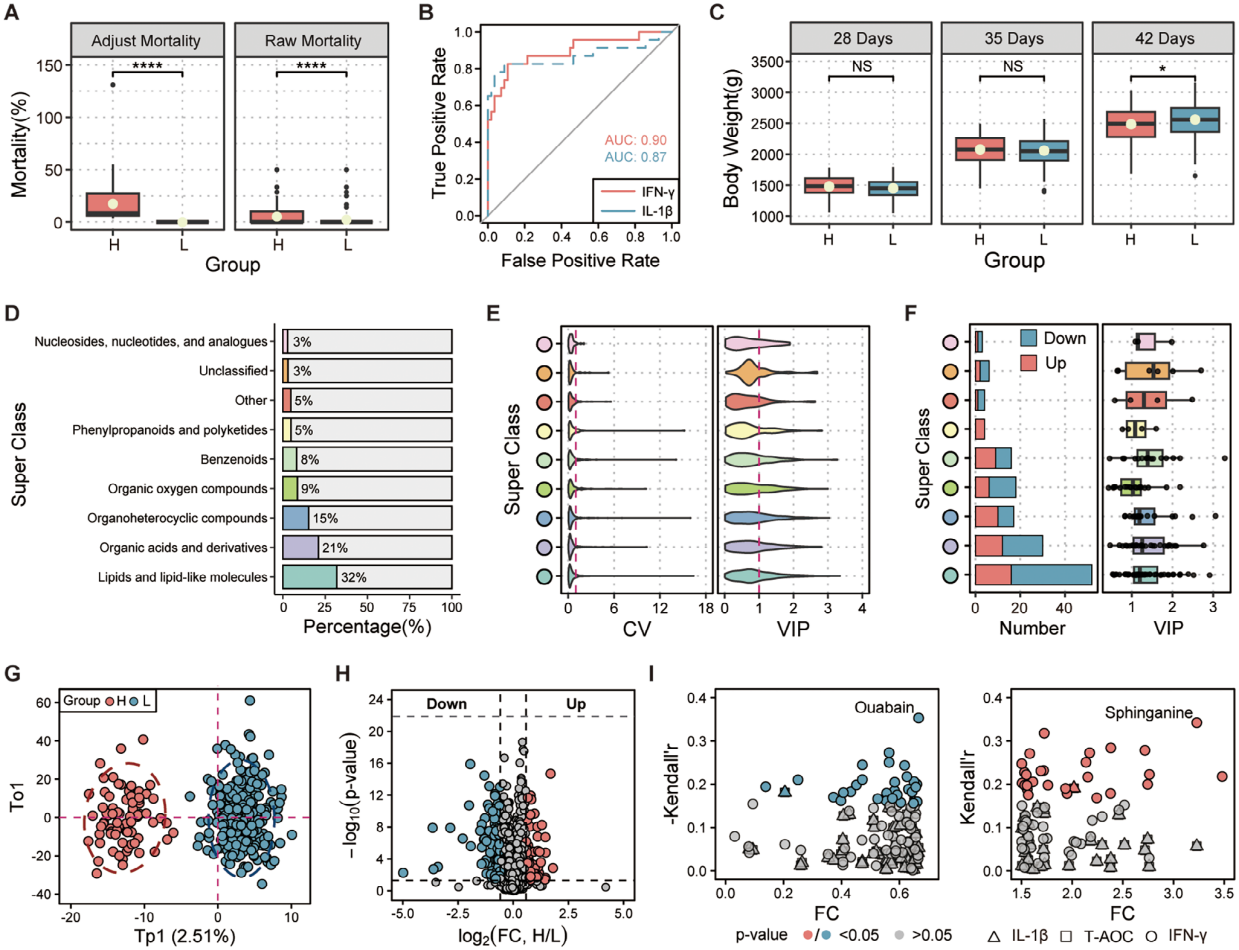
Mortality‐driven artificial selection reprograms serum metabolic networks for resource reallocation. A) Mortality difference between H and L groups in Cohort 1. Significance was determined by the Wilcoxon test, **** *P* < 0.0001. B) Receiver operating characteristic (ROC) curve analysis evaluating the diagnostic potential of interleukin‐1β (IL‐1β) and interferon‐γ (IFN‐γ) (n = 79). C) Longitudinal body weight measurements of H and L groups at 28, 35, and 42 days of age. Significance of differences between groups at each time point was determined by the Wilcoxon test, ^*^ *P* < 0.05. D) Taxonomic classification of annotated metabolites in Cohort 1 (categories and proportions). E) Coefficient of variation (CV) and variable importance in projection (VIP) across all metabolites. F) Characteristics of differentially accumulated metabolites (DAMs). Histogram showing the number of significant DAMs across superclasses: up‐regulated (red) and down‐regulated (blue). Boxplot of VIP score distribution of DAMs within each superclass. G) Orthogonal partial least squares discriminant analysis (OPLS‐DA) of metabolomes. H) Volcano plot displaying differential metabolites (red: up‐regulated, blue: down‐regulated). I) Distribution of Kendall's tau correlation coefficients between DAMs and the levels of IL‐1β, IFN‐γ, and total antioxidant capacity (T‐AOC) (n = 77). Left panel: down‐regulated DAMs. Right panel: up‐regulated DAMs.

To investigate the genetic basis of mortality, we established a continuous selection experiment by tracking 42‐day mortality across full‐sib families (**Figure** **1**). We selectively mated generation 0 (G_0_) birds from pedigrees with divergent full‐sib mortality (sire: 8.5%, dam: 9.3% vs sire: 1.5%, dam: 1.5%) (Figure S1A, Supporting Information) to produce 181 G_1_ full‐sib families, which formed our discovery cohort (Cohort 1). Based on family‐level mortality, the G_1_ progeny were stratified into high‐ (H; n = 115) and low‐mortality (L; n = 406) groups, with exhibited significantly different mortality rates (17% vs 0%) (**Figure** **2**A; Figure S2, Supporting Information). Subsequent iterative assortative mating of G_1_ birds generated an internal validation cohort (n = 102; Cohort 2), which comprised subgroups with a gradient of mortality risk (C, T1, T2, and T3) (Figure S1B, Supporting Information).

Leveraging this multi‐generational model, we designed a three‐cohort analytical workflow to identify, validate, and explore the translational relevance of mortality‐associated biomarkers. First, in the discovery cohort (Cohort 1), we compared the metabolic landscapes between the H and L groups and investigated the genetic regulation and transcriptional activity of metabolomic quantitative trait locus (mQTL). This analysis identified a panel of 16 metabolite biomarkers for chicken mortality risk, and we subsequently evaluated their predictive performance in Cohort 2. To assess the cross‐species relevance of these biomarkers, we then evaluated their conservation and predictive power using metabolomic data from a human extreme‐longevity cohort (Cohort 3, including Verify 1 and Verify 2 sub‐cohorts) and other disease datasets. Finally, we highlighted two evolutionarily conserved pathways: butyrate‐mediated microbiota‐host interactions and L‐cysteine's dual antioxidant mechanisms.

### 2.2 Serum Metabolic Rewiring in Response to Mortality‐Driven Artificial Selection

Artificial selection against mortality drove a distinct physiological trade‐off in Cohort 1. The H group mounted a stronger inflammatory response, evidenced by 1.3‐ and 1.1‐fold higher interleukin‐1β (IL‐1β) and interferon‐γ (IFN‐γ) levels, respectively, compared to the L group (Figure S3A, Supporting Information). These cytokines were not only robust predictors for mortality risk (area under the curve (AUC): 0.87–0.90) (Figure 2B), but also correlated with growth impairment in the H group. This heightened immunity came at a developmental cost. Biologically, elevated IL‐1β can impair insulin‐like growth factor I (IGF‐1)‐driven muscle growth, while IFN‐γ can increase oxidative stress, collectively diverting energetic resources from growth toward immune surveillance. Consequently, the L group, with its more tempered immune response, exhibited superior growth, achieving a 3.02% higher final body weight (Figure 2C) and a 23.33% greater weight gain (Figure S3B, Supporting Information). This pattern is consistent with an evolutionary strategy that prioritizes survival over development under high‐risk conditions.

In parallel with these physiological shifts, the selection pressure profoundly reshaped the serum metabolome of Cohort 1 (Figure S4A,B, Supporting Information). Our analysis detected 1871 metabolites spanning 9 super classes (n = 654) (Figure 2D). To probe the stability of the metabolic network, we examined the coefficient of variation (CV) for each metabolite. Intriguingly, the L group's metabolome was more variable on average (mean CV: 58.04% vs 51.38%) (Figure S4C, Supporting Information), and contained a slightly greater proportion of highly unstable metabolites (CV > 100%, 11.81% vs 10.85%) compared to the H group (Figure S4D, Supporting Information). This suggests a potential for enhanced pathway‐level buffering or substrate robustness, a phenomenon observed in metabolic networks under stress where selection may redistribute rather than amplify metabolite fluctuations.^[^ ^16^, ^17^ ^]^

To identify the specific metabolites driving these differences, we first employed orthogonal partial least squares discriminant analysis (OPLS‐DA), which revealed a clear metabolic separation between the H (n = 79) and L (n = 315) groups (R^2^Y = 0.89, Q^2^Y = 0.69) (Figure 2G). The model's robustness was confirmed by permutation testing, and 593 metabolites (32%) were identified as significant contributors (variable importance in projection (VIP) > 1) (Figure 2E). We then pinpointed 150 differentially accumulated metabolites (DAMs) in Cohort 1 (Figure 2H), with the top 50 showing highly consistent trends between the groups (Figure S5, Supporting Information). Notably, 74% of these DAMs (111 out of 150) had VIP scores exceeding 1 (Figure 2F). To assess whether specific metabolic alterations were associated with the enhanced inflammatory response in the H group, we correlated the DAMs with cytokine levels (IL‐1β and IFN‐γ) and total antioxidant capacity (T‐AOC). This correlation analysis revealed 30 down‐regulated metabolites that were negatively correlated with cytokine levels. Among these, ouabain (Kendall's r = ‐0.34, *P* = 2.46 × 10^−5^) (Figure 2I), an anti‐inflammatory cardiotonic steroid,^[^ ^18^ ^]^ was notably depleted, a change that may potentiate NF‐κB‐mediated inflammation.^[^ ^19^ ^]^ Conversely, 28 up‐regulated metabolites showed positive correlations with pro‐inflammatory cytokines. For instance, sphinganine (Kendall's r = 0.35, *P* = 5.53 × 10^−6^) was significantly accumulated (Figure 2I), which may contribute to NLRP3 inflammasome activation and IL‐1β secretion.^[^ ^20^ ^]^ Collectively, these metabolic shifts delineate a pro‐inflammatory metabolome that underlies the heightened inflammatory phenotype observed in the H group.

Kyoto encyclopedia of genes and genomes (KEGG) pathway (Figure S6A, Supporting Information) and metabolite set (Figure S6B, Supporting Information) enrichment analysis of these metabolites provided a mechanistic link to the observed phenotype (Table S1, Supporting Information). While lipids were abundant among the discriminating metabolites (18% of VIP > 1) (Figure 2E), they were disproportionately represented among the DAMs (35%) (Figure 2F), consistent with patterns of non‐specific lipid remodeling under stress.^[^ ^21^ ^]^ Crucially, pathways related to galactose metabolism and adrenergic signaling in cardiomyocytes emerged as the most significantly perturbed, aligning directly with the growth and cardiac impairments characteristic of high‐mortality broilers. Collectively, these findings provide evidence for a trade‐off between immunity and growth, supporting the thrifty phenotype hypothesis, wherein metabolic resources are reallocated to stress responses at the expense of development.^[^ ^22^, ^23^ ^]^

### 2.3 mQTLs of Mortality‐Risk Metabolites are Enriched in Liver‐Specific Regulatory Elements

**Figure 3:**
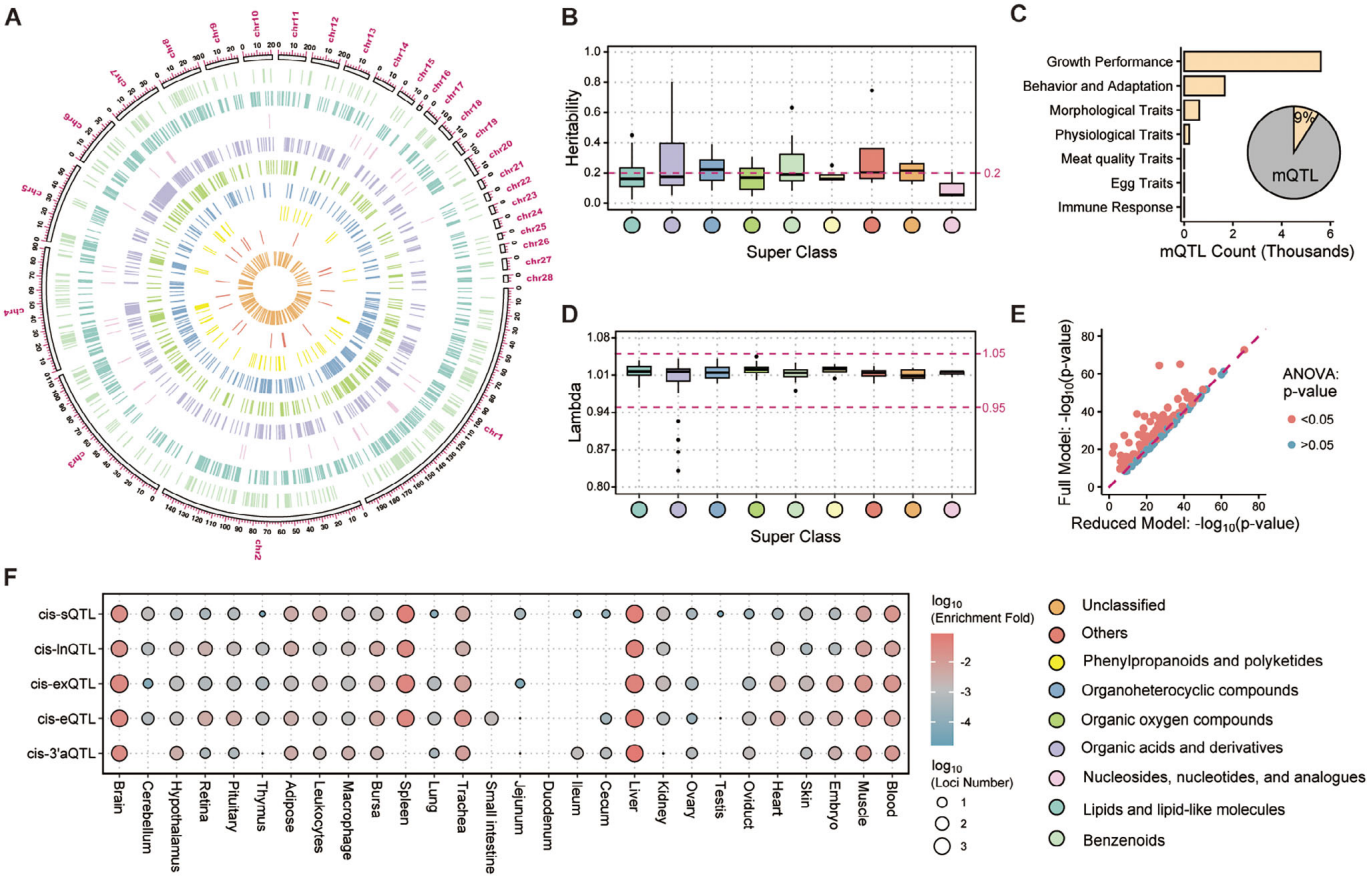
Hierarchical liver‐centric regulatory architecture mediates metabolic canalization under mortality selection. A) Genome distribution of significant SNPs (*P* < 1.69 × 10^−6^) associated with 150 differentially accumulated metabolites (DAMs). Concentric circles represent metabolite superclasses from outer to inner rings: Benzenoids, Lipids and lipid‐like molecules, Nucleosides, nucleotides and analogues, Organic acids and derivatives, Organic oxygen compounds, Organoheterocyclic compounds, Phenylpropanoids and polyketides, Others, and Unclassified. B) Distribution of the heritability of nine super class DAMs. C) Number of metabolite quantitative trait loci associated with complex traits in chickens. D) Genomic inflation factor (λ) of nine super class DAMs genome‐wide association studies (GWAS). E) Scatter plots of *P* from the reduced and full models. Points in red indicate full models significantly different from reduced models; points in blue indicate no significant difference. F) Overlap between metabolite quantitative trait loci related to DAMs identified by GWAS and molecular QTLs (molQTLs) from the Chicken GTEx.

To uncover the genetic architecture underlying the observed metabolic shifts, we performed mQTLs mapping for the 150 DAMs, a substantial fraction of which showed moderate‐to‐high heritability (43% with h^2^ > 0.2) (**Figure** **3**B). This analysis identified 45,585 mQTLs (Figure 3A), and as anticipated, metabolites with higher heritability were linked to a greater number of mQTL (Spearman's r = 0.23, *P* < 2.2 × 10^−16^). A global analysis revealed that these mQTL possess features characteristic of functional regulatory variants: compared to non‐mQTL, they exhibited significantly lower minor allele frequency (MAF; Figure S8A, Supporting Information), higher evolutionary conservation scores (phastCons; Figure S8B, Supporting Information), closer proximity to transcription start sites (TSSs; Figure S8C, Supporting Information), and the strongest enrichment in transcription flanking weak (TxFlnkWk) regions (Figure S8F, Supporting Information). On average, these loci explained 0.04% of the phenotypic variation (up to 22.87% for Isoputreanine). The robust genomic control (97% metabolites with λ = 0.95–1.05, Figure 3D) and elevated functional potential (11.46% loci with chicken combined annotation‐dependent depletion (chCADD) >10, peak score = 44.55; Figure S8E, Supporting Information) further underscore the reliability of our findings.

We next assessed the functional impact of these loci and found that 49.65% (22,634) demonstrated pleiotropic effects, each significantly associated with at least two metabolites. A striking example was observed on chromosome 1, where 592 pleiotropic loci showed associations with 60 distinct metabolites (Figure S9, Supporting Information). Notably, effect sizes varied substantially even among pleiotropic loci. For example, locus chr1:150017622 was significantly associated with four metabolites, including Myxin (mFC = ‐2.21) and Valerylcarnitine (mFC = 0.16), yet exhibited markedly different directions and magnitudes of effect across them (Figure S8G, Supporting Information). These pleiotropic mQTLs were annotated to genes significantly enriched for hepatic functions, such as ABC transporters and lipid metabolism, pointing to the liver as a key regulatory hub (Figure S10, Supporting Information). Moreover, 4,115 of these mQTLs overlapped with known QTL for complex production traits like growth and reproduction, directly linking these metabolic regulators to whole‐animal physiology (Figure 3C; Figure S11, Supporting Information). For instance, the intronic variant chr4:75016308 within the *SLIT2* gene exemplifies pleiotropy. This variant influences multiple metabolite levels and is significantly associated with 20 production traits, such as body weight and average daily gain. Mechanistically, *SLIT2* encodes an osteokine that mediates a bone‐fat signaling axis, suggesting profound integration of metabolic control and developmental processes.^[^ ^24^ ^]^

Given that the vast majority of DAM‐associated mQTLs were non‐coding (e.g., 48% intronic, 21% intergenic; Figure S8D, Supporting Information), we performed a systematic intersection analysis with five types of molQTLs from the Chicken GTEx to assess their potential effects on gene regulatory processes. These mQTLs exhibited strong tissue‐specific enrichment, with the highest concentration in the liver and secondary enrichments in the spleen and brain (Figure 3F). Among the different classes of molQTL, variants affecting exon usage (cis‐exQTL) showed the highest concordance with mQTLs (24,548 overlapping loci, 53.85%), followed by expression QTL (cis‐eQTL) (13,965 overlaps, 30.64%). Building upon these broad overlaps, colocalization analysis further pinpointed 36 specific genetic variants distributed across chromosomes 2, 3, 4, 6, and 9 (PH4 > 0.5; SNP‐level PH4 > 0.5) that are shared between metabolite and molecular phenotypes (Figure S12, Supporting Information), providing strong evidence for shared causal mechanisms. Notably, 10 of these colocalized variants (27.8%) were specifically associated with liver tissue, further underscoring the liver's pivotal role as a metabolic regulatory hub. To further dissect this within the hepatic context, we found that variants involved in mQTL‐cis‐exQTL overlaps corresponded to the largest number of differentially expressed genes (Figure S13, Supporting Information). Collectively, these findings delineate a hierarchical regulatory architecture wherein genetic variation influencing exon‐level processing appears to be a predominant mechanism driving metabolic remodeling in the liver. This mechanistic link is exemplified by the shared variant rs315746842, which is associated with sakacin A levels (acting as an mQTL) and concurrently functions as a cis‐exQTL for the *NRF2* gene in liver tissue. *NRF2*, encoding a master regulator of antioxidant responses,^[^ ^25^, ^26^, ^27^ ^]^ was itself differentially expressed in the liver between the H and L groups. Thus, the overlap of rs315746842 with both mQTL and cis‐exQTL signals strongly suggests that genetic variation affecting *NRF2* exon usage may contribute to the regulation of sakacin A metabolism, potentially via *NRF2*‐mediated antioxidant pathways influencing sakacin A turnover or function within the liver.

### 2.4 Disentangling the Genetic and Physiological Drivers of Metabolic Divergence

To determine whether the metabolic differences between the H and L groups were driven primarily by underlying genetic variation or by the physiological state associated with mortality risk, we first confirmed that the general genetic landscape was comparable between groups, with no significant differences in MAF (Figure S14, Supporting Information) and consistent heritability patterns (Figure S15, Supporting Information). Next, to quantify the relative contributions to metabolic variation, we performed variance decomposition. We observed that the top mQTL were indeed the largest single contributors (explaining a mean of 6.56% of the variance), exceeding the direct effects of mortality status (5.76%) and body weight (1.69%) (Figure S16A, Supporting Information). Nevertheless, the fact that the top mQTL explained only a modest portion of the total variance indicated that a substantial portion of the metabolic divergence was not explained by genetics alone, as evidenced by the significantly different abundances of metabolites associated with identical genotypes between the H and L groups (Figure S16B, Supporting Information). To formally disentangle these genetic and physiological effects, we compared two models for each of the 150 DAMs: one accounting only for the top mQTL (Reduced model) and another including both the mQTL and the mortality group status (Full model). The analysis revealed a clear result: incorporating the mortality group as a factor significantly improved the model fit for 49 DAMs (Figure S17A, Supporting Information), and a significant group effect was detected for a majority of the metabolites tested (110 out of 150, *P* < 0.05; Figure 3E). Collectively, these findings indicate that while genetic predispositions provide a substantial foundation for metabolic profiles, the physiological state associated with mortality risk is a dominant force that actively remodels the metabolome (Figure S17B, Supporting Information). This exemplifies the concept of metabolic canalization, where a biological network buffers underlying genetic variation while channeling a robust and adaptive response to a strong physiological stressor.^[^ ^28^, ^29^, ^30^ ^]^

### 2.5 16‐Metabolite Biomarker Panel Identified by Integrating Machine Learning and Network Analysis Predicts Mortality Risk

**Figure 4:**
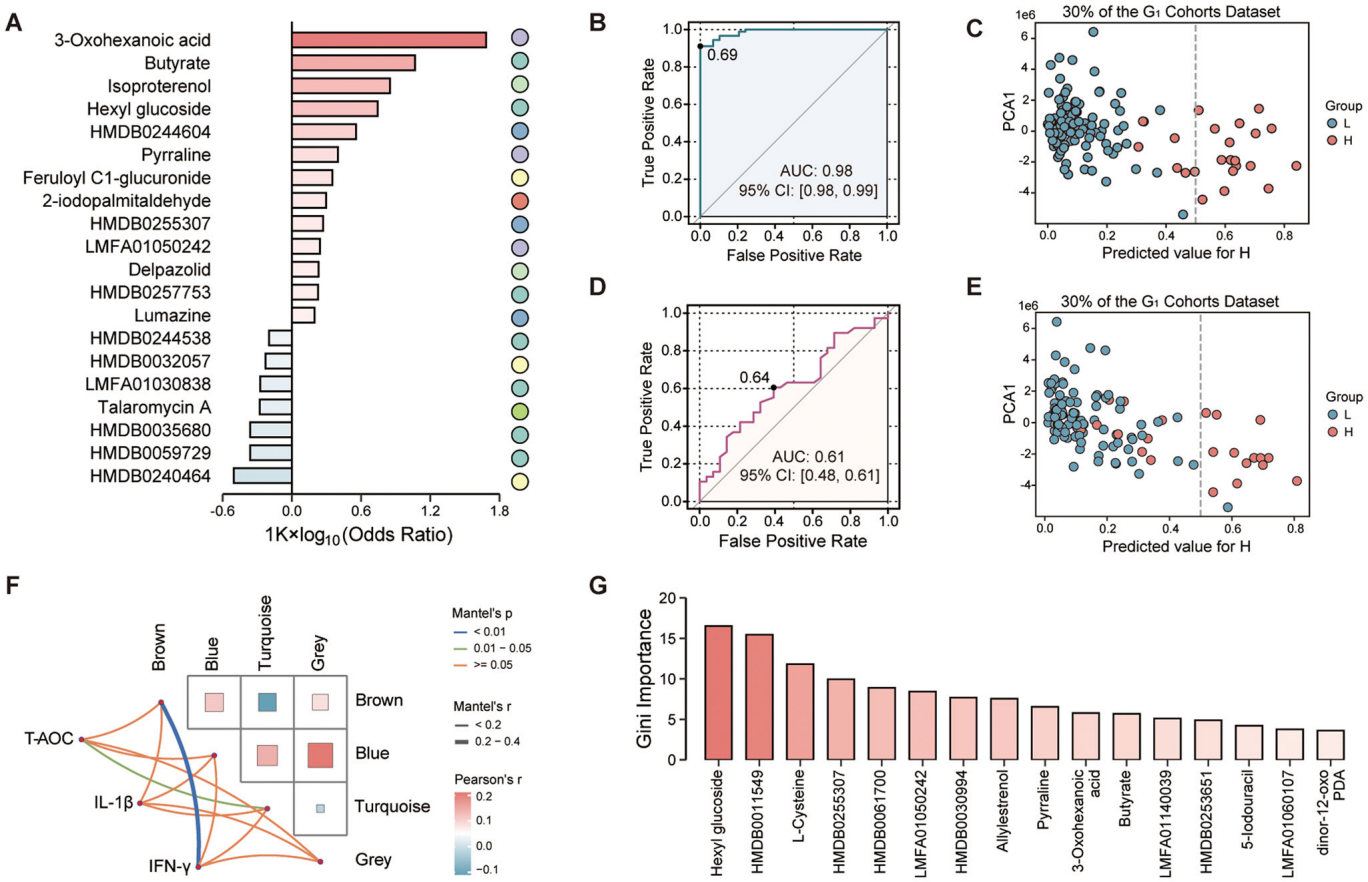
Integrative machine learning and network analysis identified biomarkers predictive of mortality risk. A) Distribution of odds ratio (OR) for the top 20 candidate key metabolites selected by the least absolute shrinkage and selection operator (LASSO) model. B) ROC curve analysis of the random forest (RF) model constructed with 92 key metabolites using 30% of Cohort 1 as the test set. C) Predictive performance of the RF model (constructed with the 92 key metabolites) in discriminating H (red) versus L (blue) groups in the held‐out test set of Cohort 1. D) ROC curve analysis of the RF model (constructed with the 92 key metabolites) applied to the low mortality (C) and high mortality (T3) groups in Cohort 2. E) Predictive performance of the RF model (constructed with the 16 metabolic biomarkers) in discriminating H (red) versus L (blue) groups in the Cohort 1 test set. F) Heatmap illustrating relationships between metabolite modules and total antioxidant capacity (T‐AOC), IL‐1β, and IFN‐γ levels (n = 77). G) RF variable importance ranking of the 16 metabolic biomarkers. The color of the bars changes from dark to light to indicate decreasing importance.

To identify a robust set of metabolic biomarkers for mortality risk, we implemented a multi‐stage computational strategy. First, we applied least absolute shrinkage and selection operator (LASSO) regression with 10‐fold cross‐validation in Cohort 1 (Figure S18, Supporting Information) and identified 92 candidate metabolites linked to mortality risk (**Figure** **4**A; Table S2, Supporting Information). A random forest (RF) model trained on these 92 features achieved excellent predictive performance in the internal test set (AUC: 0.98; Figure 4B). Using a classification threshold of 0.5, the RF model accurately identified samples from the L group in the internal test set (Figure 4C). However, this model's performance dropped significantly in the external validation cohort (Cohort 2; AUC: 0.61) (Figure 4D), indicating a propensity for overfitting. To overcome this, we sought to integrate the biological context of cytokines using WGCNA on the full metabolome (Figure S19A–C, Supporting Information). This analysis clustered metabolites into four modules, with the “brown” module (219 metabolites) showing a significant positive correlation with IFN‐γ levels (Pearson's = 0.32, *P* = 0.02) (Figure 4F; Figure S19D–F, Supporting Information), a key cytokine identified in our earlier analysis. By intersecting the 92 LASSO‐selected metabolites with the 219 biologically relevant metabolites in the brown module, we distilled a high‐confidence panel of 16 core biomarkers (Figure S20A and Table S3, Supporting Information), including hexyl glucoside, butyrate, and L‐cysteine. These candidates span three major chemical super‐classes: lipids and lipid‐like molecules (n = 8), organic acids and derivatives (n = 4), and organoheterocyclic compounds (n = 4). Among these, butyrate^[^ ^31^ ^]^ and L‐cysteine^[^ ^32^ ^]^ possess well‐documented antioxidant and anti‐inflammatory properties, while LysoPC(18:2)^[^ ^33^ ^]^ is a known pro‐inflammatory mediator. Pyrraline has been shown to activate the Nrf2 pathway,^[^ ^34^ ^]^ suggesting a potential role in antioxidant responses.

This refined 16‐metabolite signature proved to be both robust and highly predictive. A new RF model trained exclusively on these 16 biomarkers demonstrated strong predictive power in Cohort 1 (99% accuracy in classifying L‐group individuals; Figure 4E). Individually, all 16 metabolites showed predictive value (AUC ≥ 0.50; Figure S20C, Supporting Information). Hexyl glucoside, identified as the most significant metabolite by RF feature importance ranking (Figure 4G), achieved the highest AUC of 0.80. Furthermore, hexyl glucoside displayed strong positive correlations with butyrate (Pearson's r = 0.53, *P* < 2.20 × 10^−16^) and L‐cysteine (Pearson's r = 0.37, *P* = 1.70 × 10^−14^) (Figure S21, Supporting Information), implying potential metabolic co‐regulation. Ten of these biomarkers were positively correlated with mortality risk, while three were negatively correlated (Figure S20B, Supporting Information). Most biomarkers also exhibited low variability (CV: 41.20% vs 60.91% of population mean) and high discriminatory power (VIP: 1.87 vs 0.84 of the above mean), underscoring their stability and suitability as a predictive signature (Figure S20D, Supporting Information).

### 2.6 Conserved Immune Coupling of Mortality Biomarkers in Chickens and Humans

**Figure 5:**
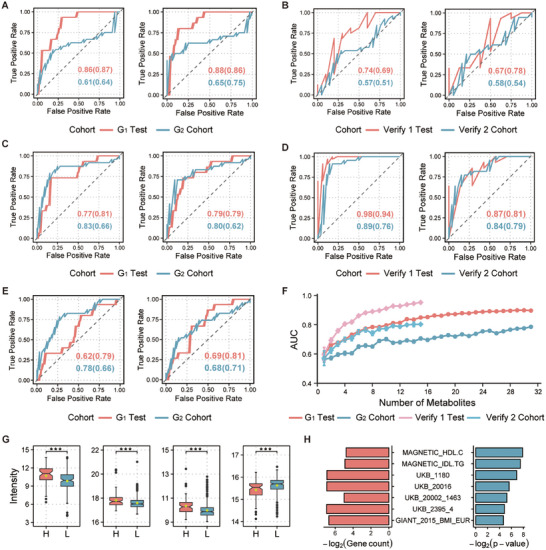
Acute mortality biomarkers conserved across species exhibit divergent immune‐metabolic circuitry. A) Synergistic immune and metabolic profiling enhances precision mortality forecasting in chickens. ROC curves of the prediction model constructed with 16 metabolic biomarkers in the Cohorts 1 and 2 (left). ROC curves of the prediction model after adding immune‐related signature metabolites to the 16‐metabolite model in the Cohorts 1 and 2 (right). B) Cross‐species validation of chicken mortality biomarkers in a human extreme‐longevity cohort. ROC curves of the baseline prediction model using three chicken‐derived metabolic biomarkers (left). ROC curves after augmenting the model with immune‐related biomarkers (right). C) Immune‐metabolic synergy enhances mortality risk prediction in chickens. ROC curves of the prediction model constructed with three metabolic biomarkers in the Cohorts 1 and 2 (left). ROC curves of the prediction model after adding immune‐related signature metabolites to the three‐metabolite model in Cohorts 1 and 2 (right). D) Mortality prediction in a human extreme‐longevity cohort comparative efficacy of longevity‐associated versus acute pathology‐associated metabolic biomarkers. ROC curves for human mortality risk prediction using longevity‐associated biomarkers (left). ROC curves for human mortality risk prediction using acute pathology‐associated biomarkers (right). E) Cross‐species validation of human‐derived metabolic signatures in broiler mortality prediction. ROC curves for mortality risk prediction in chickens using human longevity‐associated biomarkers (left). ROC curves for mortality risk prediction in chickens using human acute pathology‐associated biomarkers (right). F) Conserved dose‐dependent enhancement of mortality prediction across species. Increasing numbers of signature metabolites improve mortality risk prediction accuracy in both chicken and human cohorts. G) Differential accumulation of mortality‐associated metabolites between H and L groups. Box plots show that risk‐elevating metabolites (N1‐acetylspermidine, L‐acetylcarnitine, 21‐deoxycortisol) are significantly elevated in the H group, while the risk‐lowering metabolite pseudouridine is elevated in the L group. Significance was determined by the Wilcoxon test, ^***^ *P* < 0.001. H) Enrichment analysis of metabolic biomarker‐associated genes across 114 human disease‐related gene clusters.

To investigate the translational relevance of our 16 mortality biomarker panel, we assessed its ability to predict mortality risk in humans. The signature demonstrated considerable predictive power in both broiler cohort (AUC = 0.77) and human cohort (AUC = 0.74) (**Figure** **5**B,C), highlighting a conserved metabolic basis for mortality risk. Notably, the directionality of association for individual metabolites with mortality was consistent across species. For instance, metabolites associated with increased mortality risk in chickens, such as N1‐acetylspermidine and L‐acetylcarnitine, are known to accumulate in human metabolic disorders, including insulin resistance and cardiovascular disease (Figure 5G).^[^ ^35^ ^]^ Conversely, metabolites associated with survival in chickens, such as pseudouridine, have been linked to protective metabolic phenotypes in humans (Figure 5G).^[^ ^35^ ^]^

Furthermore, we observed a potential divergence in immuno‐metabolic interactions between the species. Incorporating a subset of metabolites previously associated with cytokine activity enhanced the predictive performance of the model in chickens (e.g., AUC increased from 0.77 to 0.79 for the 3‐metabolite model and from 0.86 to 0.88 for the 16‐metabolite model) (Figure 5A,C), but paradoxically reduced its performance in the human cohort (AUC decreased from 0.74 to 0.67) (Figure 5B). This finding may suggest a more pronounced and critical coupling between specific metabolic pathways and the immune system in the pathophysiology leading to mortality in broilers, potentially reflecting their unique physiological pressures from rapid growth.^[^ ^36^, ^37^, ^38^, ^39^ ^]^ To further probe this distinction, we tested two human‐derived biomarker models on the chicken cohort. A model built from biomarkers of acute organ failure in humans predicted chicken mortality with significantly greater accuracy (AUC = 0.69) than a model based on biomarkers of human longevity (AUC = 0.62) (Figure 5E), which contrasts with their relative performances in the human cohort where the longevity model outperforms the organ failure model (Figure 5D). This reinforces the notion that the conserved signature reflects mechanisms of acute physiological collapse rather than chronic aging. Moreover, across both species, we observed that increasing the number of signature metabolites in the predictive models led to a consistent improvement in performance, indicating a synergistic effect where the combined panel provides a more comprehensive snapshot of the pathophysiological state underlying mortality risk (Figure 5F).

To uncover the genetic basis for this cross‐species conservation, we analyzed the 220 genes associated with the 16 biomarkers, among which 11 were linked to higher mortality risk and two linked to lower risk (Figure S23B, Supporting Information). These genes were enriched in conserved biological functions, including lipid metabolism and insulin signaling (Figure S24, Supporting Information). Notably, 15 were direct orthologs of human loci implicated in metabolic syndrome (e.g., *MAP2K5*, *LIPC, NFAT5*) (Figure S23A, Supporting Information).^[^ ^40^ ^]^ Probabilistic transcriptome‐wide association studies (PTWAS) further linked the chicken genetic architecture to seven human complex traits, including lipid levels, body mass index, and circadian rhythm (Figure 5H).^[^ ^41^ ^]^ By leveraging causal metabolite‐disease databases, we confirmed that homologous metabolites influence key human clinical phenotypes; for instance, cysteine derivatives showed causal links to rheumatoid arthritis and neurodegenerative pathologies, while specific Lysophosphatidylcholines were linked to type 2 diabetes and arteriosclerosis (Figure S25, Supporting Information).^[^ ^42^ ^]^ Collectively, these results establish an evolutionarily conserved framework in which mortality‐associated biomarkers and their genetic regulators reflect shared pathogenic mechanisms underlying metabolic dysregulation across species.

### 2.7 Host Genetics and Gut Microbiota Jointly Shape Butyrate‐Mortality Association

**Figure 6:**
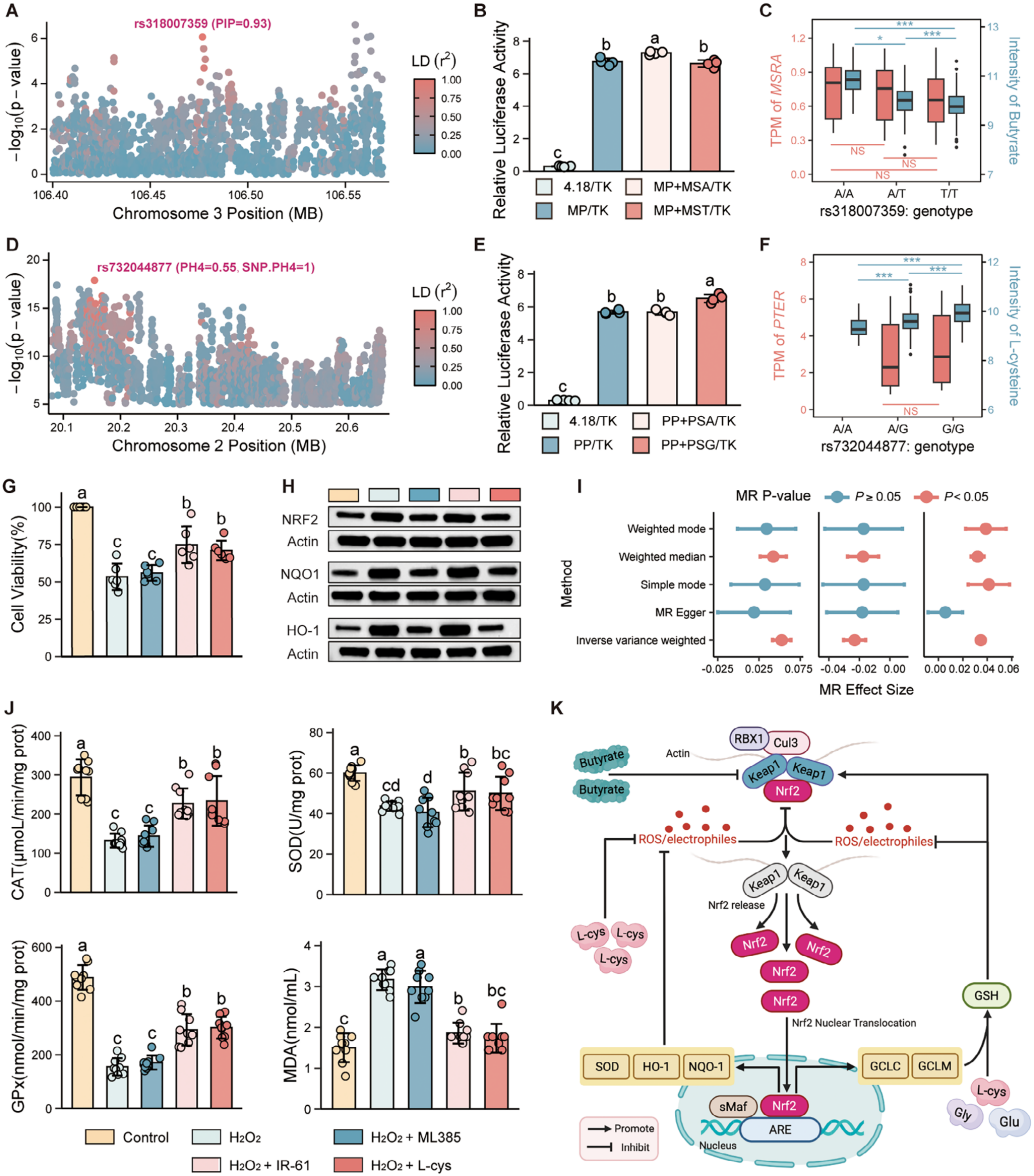
Integrated multi‐omics reveals butyrate‐L‐cysteine associated genetic loci and their antagonism on the Keap1/Nrf2 pathway. A) Manhattan plot highlighting the candidate genomic region identified by GWAS for butyrate. B) Regulatory effect of the intronic SNP rs318007359 (A or T alleles) on the activity of the *MSRA* promoter using a luciferase reporter assay (n = four biological replicates). TK: pRL‐TK (internal control); 4.18: empty vector pGL4.18; MP: pGL4.18 with the wild‐type *MSRA* promoter fragment; MSA: pGL4.18 with the *MSRA* promoter fragment containing the A allele; MST: pGL4.18 with the *MSRA* promoter fragment containing the T allele. Values are expressed as the mean ± standard error. Data were analyzed by one‐way ANOVA followed by Fisher's LSD test. Different letters indicate significant differences (*P* < 0.05). C) Boxplots of *MSRA* expression levels (red) in heart, liver, and spleen tissues (n=16) and butyrate intensity (blue) in serum (n = 623), each stratified by genotype at SNP rs318007359. Significance was determined by the Wilcoxon test, NS *P* > 0.05, ^*^ *P* < 0.05, ^***^ *P* < 0.001. D) Manhattan plot highlighting the candidate genomic region identified in GWAS for L‐cysteine. E) Regulatory effect of the intronic SNP rs732044877 (A or G alleles) on the activity of the *PTER* promoter using a luciferase reporter assay (n = four biological replicates). TK: pRL‐TK (internal control); 4.18: empty vector pGL4.18; PP: pGL4.18 with the wild‐type *PTER* promoter fragment; PSA: pGL4.18 with the *PTER* promoter fragment containing the A allele; PSG: pGL4.18 with the *PTER* promoter fragment containing the G allele. Values are expressed as the mean ± standard error. Data were analyzed by one‐way ANOVA followed by Fisher's LSD test. Different letters indicate significant differences (*P* < 0.05). F) Boxplots of *PTER* expression levels (red) in heart and spleen tissues (n = 17) and L‐cysteine intensity (blue) in serum (n = 626), each stratified by genotype at SNP rs732044877. Significance was determined by the Wilcoxon test, NS *P* > 0.05, ^*^ *P* < 0.05, ^***^ *P* < 0.001. G) Cell viability of HD11 cells under different treatments, determined by CCK‐8 assay (n = three biological replicates). Values are expressed as the mean ± standard error. Data were analyzed by one‐way ANOVA followed by Fisher's LSD test. Different letters indicate significant differences (*P* < 0.05). H) Nrf2, NQO1, and HO‐1 protein levels under different treatments (Western blot, n = three biological replicates). I) Mendelian randomization (MR) reveals causal effects of cecal *Blautia* abundance (left), *Christensenellaceae_R‐7_group* abundance (middle), and the *Blautia*/*Christensenellaceae_R‐7_group* (B/C) ratio (right) on serum butyrate levels. J) Levels of oxidative status indices across different treatments (n = three biological replicates). CAT; antioxidants including catalase; GPx: glutathione peroxidase; SOD: superoxide dismutase; and MDA: malondialdehyde. Values are expressed as the mean ± standard error. Data were analyzed by one‐way ANOVA followed by Fisher's LSD test. Different letters indicate significant differences (*P* < 0.05). K) Dual Nrf2‐dependent and ‐independent antioxidant pathways mediated by butyrate and L‐cysteine under stress conditions. Image created with BioRender.com, and published with permission.

We conducted an in‐depth investigation of two representative metabolites from the panel: butyrate and L‐cysteine. Butyrate, a gut microbiota‐derived short‐chain fatty acid that is critical for immune homeostasis,^[^ ^43^, ^44^ ^]^ was consistently elevated in high‐mortality individuals across both Cohort 1 (Kendall's r = 0.31, *P* = 4.55 × 10^−4^) and validation Cohort 2 (Figure S26A,B; Supporting Information). To pinpoint the host genetic determinants, an mGWAS analysis identified 17 candidate loci within chr3:101.5–104.3 Mb. Fine‐mapping of this region subsequently pinpointed an intronic variant, rs318007359, as the likely causal variant (posterior inclusion probability (PIP) = 0.93) (**Figures** **6**A; S27A,B, Supporting Information). Functional validation confirmed that the “A” allele of rs318007359 significantly enhanced the activity of its promoter (*P* < 0.05, dual‐luciferase assay), consistent with elevated *MSRA* expression in A/A homozygotes across multiple tissues (Figure 6B,C). This functional effect translated directly into the mortality phenotype: the high‐risk “A” allele frequency was twice as high in the H group (34%) as the L group (17%), and *MSRA* expression positively correlated with butyrate levels (Figure S33A–C, Supporting Information). Mendelian randomization (MR) analysis further established a causal relationship between cecal and systemic serum butyrate levels (β [standard error, SE] = 0.16 [0.07], *P* = 0.03) (Figures S28A,B and S29, Supporting Information), supporting the biological pathway from gut microbial production to systemic circulation.

We next investigated whether this host‐driven elevation in butyrate was associated with a corresponding shift in the gut microbiota, using previously collected datasets.^[^ ^45^ ^]^ While alpha‐diversity was comparable between groups (Figure S30B, Supporting Information), beta‐diversity analysis revealed a clear and significant separation of cecal microbial communities based on mortality risk (Figure S30A, Supporting Information). At the genus level, *Blautia*, a known butyrate producer, was 1.82‐fold enriched in the high‐butyrate (HB) group, whereas *Christensenellaceae_R‐7_group* was depleted (Figure S30C,D; Supporting Information). Consistent with this group‐wise difference, correlation analysis at the individual level confirmed that elevated butyrate levels were associated with a distinct microbial signature, characterized by significant enrichment of *Blautia* (Kendall's r = 0.21, *P* = 1.9× 10^−3^) (Figure S31A, Supporting Information) and depletion of *Christensenellaceae_R‐7_group* (Kendall's r = ‐0.29, *P* = 1.5 × 10^−5^) (Figure S31B, Supporting Information). Based on these associations, we constructed a ratio of beneficial *Blautia* to depleted *Christensenellaceae_R‐7_group* (B/C) as a potential robust indicator of the pro‐butyrate microbial environment, and assessed its predictive performance. The resulting B/C ratio emerged as a powerful microbial biomarker, outperforming either taxon alone (AUC = 0.82) (Figure S31D, Supporting Information) and strongly correlating with butyrate levels (Kendall's r = 0.36, *P* = 1.3 × 10^−7^) (Figure S31C, Supporting Information). To further evaluate the causal relationship of these associations, we employed MR analysis. The results demonstrated that *Blautia* (β [SE] = 0.05 [0.01], *P* = 3.58 × 10^−20^), *Christensenellaceae_R‐7_group* (β [SE] = ‐0.02 [0.004], *P* = 3.93 × 10^−10^), and the B/C ratio (β [SE] = 0.04 [0.01], *P* = 3.69 × 10^−6^) each exerted causal effects on cecal butyrate production (Figure 6I; Figure S28C–H, Supporting Information), supporting a microbiota‐to‐butyrate causal direction. This integrated evidence connects the entire axis from host genetics to microbial ecology: carriers of the high‐risk *MSRA* “A” allele exhibited not only elevated butyrate levels but also a pro‐butyrate microbial signature, characterized by significant enrichment of *Blautia* and depletion of *Christensenellaceae_R‐7_group*. Together, these findings provide direct evidence for host genetic orchestration of a specific microbial niche linked to a metabolic phenotype and, ultimately, to mortality risk.

### 2.8 *PTER* Variant‐Mediated L‐cysteine Modulation of the Keap1/Nrf2 Antioxidant Axis

L‐cysteine, a thiol‐containing amino acid with critical roles in antioxidant defense and glutathione biosynthesis,^[^ ^32^, ^46^, ^47^ ^]^ whose accumulation was correlated with mortality risk (Kendall's r = 0.37, *P* = 3.18 × 10^−5^) and was consistently elevated in the high‐mortality group (Figure S26C,D, Supporting Information). Following an mGWAS for L‐cysteine (Figure S27C,D, Supporting Information), fine‐mapping and colocalization analysis(Figure S32, Supporting Information) of the associated loci revealed that the intronic variant rs732044877 in *PTER* emerged as a key candidate causal variant (Figure 6D). The risk‐associated “G” allele significantly enhanced the activity of its promoter in a dual‐luciferase assay (Figure 6E). Consistent with this, G/G homozygotes exhibited elevated *PTER* expression in the heart and spleen (Figure 6F; Figure S33D,F), linking this allele‐driven gene regulation to the mortality phenotype. Intriguingly, this effect was tissue‐specific, as the “G” allele was associated with reduced *PTER* expression in the liver (Figure S33E, Supporting Information), suggesting complex, context‐dependent regulation. Genotypic stratification revealed a slight enrichment of G/G homozygotes in the H group (50% vs 45% in L group).

Beyond its genetic regulation, we investigated how L‐cysteine modulates cellular antioxidant defenses. The Keap1/Nrf2 axis is a central regulator of redox homeostasis,^[^ ^48^, ^49^ ^]^ a role supported in our system by the treatment with an Nrf2 modulator (IR‐61), which increased cell viability and decreased oxidative stress (Figure 6G). We then tested the effects of L‐cysteine itself. Notably, while butyrate (also elevated in the H group) is known to activate Nrf2,^[^ ^50^ ^]^ L‐cysteine initiated a synergistic feedback mechanism. As expected, L‐cysteine treatment effectively mitigated H_2_O_2_‐induced oxidative stress, evidenced by a 45.20% decrease in malondialdehyde (MDA) levels (Figure 6J). However, the underlying mechanism revealed a clear decoupling of the Nrf2 transcriptional program from its functional enzymatic output. Specifically, L‐cysteine treatment significantly suppressed the expression of Nrf2 (29.02% decrease), quinone oxidoreductase 1(NQO1) (27.56% decrease), and heme oxygenase 1(HO‐1) (37.61% decrease) (Figure 6H; Figure S35, Supporting Information). In direct contrast, it simultaneously and potently boosted the enzymatic activities of key antioxidant enzymes: catalase (CAT; 1.76‐fold), superoxide dismutase (SOD; 1.14‐fold), and glutathione peroxidase (GPx; 1.93‐fold) (Figure 6J).

This dual regulatory strategy achieves a highly efficient metabolic equilibrium. First, L‐cysteine's free thiol group provides an immediate, non‐transcriptional defense by directly restoring oxidized enzymes to their active state.^[^ ^51^ ^]^ Second, as the rate‐limiting substrate for glutathione (GSH) synthesis, L‐cysteine elevates intracellular GSH levels. This increase provides sophisticated negative feedback to the Nrf2 pathway; GSH binds to Keap1, stabilizing the Keap1‐Nrf2 complex and promoting Nrf2 degradation, thus preventing a sustained and energetically costly transcriptional over‐activation.^[^ ^52^, ^53^ ^]^ Therefore, the combined effects of butyrate (Nrf2 activator) and L‐cysteine (Nrf2 feedback inhibitor and direct enzyme activator) create a synergistic and balanced antioxidant system^[^ ^54^, ^55^ ^]^: one metabolite primes the transcriptional potential, while the other provides rapid enzymatic action and a crucial off‐switch to maintain redox homeostasis without excessive resource expenditure (Figure 6k).

## 3 Discussion

Mortality, a critical indicator in animals breeding, is particularly when genetic determinants are ambiguous and cannot be used for selection early. This is especially valuable in the highly intensive chicken farming industry.^[^ ^56^ ^]^ This study establishes the first large‐scale serum metabolomic atlas for chicken mortality by profiling 1,871 metabolites via untargeted liquid chromatography‐mass spectrometry (LC‐MS/MS). These findings underscore the predictive potential of mortality‐associated biomarkers, though structural annotation was limited to ≈10% of the detected MS features due to current database constraints.^[^ ^57^, ^58^ ^]^ Our results demonstrate the power of metabolomics in deciphering the intricate interplay between metabolic pathways and mortality in broiler chickens. By analyzing metabolomic data from 394 individuals, we identified 150 DAMs between the H and L mortality groups, of which 74% exhibited VIP scores greater than 1, confirming their strong discriminative utility. Key metabolites included Genipin 1‐gentiobioside (linked to fatty acid oxidation) and Isoproterenol (implicated in immune regulation).^[^ ^59^, ^60^ ^]^ This aligns with prior research associating specific metabolites with mortality risk, such as elevated N1‐acetylspermidine and 21‐deoxycortisol in the H group and pseudouridine depletion in the L group.^[^ ^35^ ^]^

The driving factors behind biomarker divergence between the H and L groups underscore a fundamental resilience of metabolic phenotypes, given the comparable influence of non‐genetic factors and genetic determinants (mQTLs). This robustness can be interpreted through the framework of metabolic canalization, whereby core metabolic flux is maintained against genetic and environmental variation through buffering mechanisms within biochemical networks.^[^ ^61^ ^]^ Such buffering capacity likely reflects evolutionary optimization under intense artificial selection. Critically, this canalization operates through non‐coding regulatory architectures, as evidenced by the predominantly intronic (48%) and intergenic (21%) localization of mQTL that converge with exQTL to drive hepatic metabolic remodeling. This evolved mechanism of canalization ultimately facilitates a balance in resource allocation between growth and survival, thereby determining mortality risk. The distinct metabolic profiles observed in the H and L groups, characterized by dysregulated lipid homeostasis, redox imbalance, and cytokine‐driven immune activation, closely resemble the “thrifty phenotype” hypothesis for human metabolic syndromes.^[^ ^23^, ^62^ ^]^ Supporting this interpretation, a substantial number of mQTLs exhibit an extensive and evolutionarily conserved regulatory pattern, being enriched in repressive marks across tissues while depleted of active regulatory elements, consistent with a buffering role. This suggests potential pleiotropic metabolic regulation. Furthermore, this genome‐wide buffering paradoxically coexists with pervasive pleiotropy, as evidenced by nearly half of metabolite‐associated loci simultaneously regulating hepatic functions and diverse complex traits through a shared genetic architecture.

Our multi‐stage biomarker identification strategy, which integrated LASSO regression for statistical rigor with WGCNA for biological context, yielded a robust 16‐metabolite signature that overcomes overfitting limitations observed in conventional approaches. The signature demonstrated substantial cross‐species predictive power, achieving AUC values of 0.77 in broiler chickens and 0.74 in humans. This conserved predictive accuracy reflects evolutionary retention of core metabolic stress responses, primarily mediated through shared pathways such as the glycerolipid metabolism,^[^ ^63^ ^]^ the insulin signaling,^[^ ^64^ ^]^ and the redox regulation pathways. Mechanistic support for this conservation is provided by the orthology of the 16 mortality‐associated genes (including *LIPC* and *MAP2K5*) with human metabolic syndrome loci, coupled with their significant enrichment in the calcium signaling and the Wnt pathways.^[^ ^65^ ^]^ These pathways are fundamental to energy homeostasis across vertebrate species. Notably, the divergent effects of cytokine‐related metabolites on model performance (a gain of 0.02 AUC in chickens versus a loss of 0.07 AUC loss in humans) reveal species‐specific immune‐metabolic coupling. In broilers, acute mortality aligns more closely with human organ failure biomarkers (AUC = 0.69) than with longevity patterns (AUC = 0.62), reflecting an “inflammo‐metabolic shunt” mechanism. This process involves inflammation that catastrophically diverts metabolic resources from anabolic growth to immune activation, which is directly supported by our observations of significantly reduced body weight and elevated pro‐inflammatory cytokines in the H group compared to the L group.

This study also elucidates that host genetic variants coordinately regulate butyrate through *MSRA* and microbial interactions. The rs318007359 variant (PIP = 0.93) is located within an intron of the *MSRA* gene, which significantly boosts promoter activity, thereby driving increased *MSRA* expression, butyrate accumulation, and subsequent alterations in gut microbial ecology. Previous studies have demonstrated *MSRA*’s multifaceted protective functions: suppressing pancreatic cancer metastasis by repairing oxidized methionine residues in PKM2 and preventing atrial fibrillation by mediating antioxidant therapies.^[^ ^66^, ^67^ ^]^ Importantly, our finding that *MSRA* serves as a causal gene for butyrate metabolism provides novel evidence for its central role in regulating systemic redox homeostasis. Given its beneficial roles, we observed a paradoxical positive association between elevated systemic butyrate levels and increased mortality risk. Mendelian randomization analysis supported a causal effect of cecal butyrate on serum butyrate, suggesting that the increased serum butyrate in high‐mortality individuals may originate from gut microbial production. Furthermore, *Blautia*, *Christensenellaceae*, and their ratio (B/C ratio) were causally linked to cecal butyrate levels, indicating a potential microbiota–butyrate causal axis. The elevated systemic butyrate likely results from impaired intestinal barrier integrity (“leaky gut”),^[^ ^68^ ^]^ which may allow microbially produced butyrate to enter the circulation. We hypothesize that once in the bloodstream, butyrate might initiate compensatory antioxidant responses. However, these responses could be overwhelmed by concomitant LPS‐induced systemic inflammation and other factors,^[^ ^69^, ^70^ ^]^ which may ultimately contribute to higher mortality. These systemic effects appear to originate from, and are reflected in, distinct gut microbiota configurations: high‐butyrate/high‐mortality groups were enriched in *Blautia*, while low‐butyrate groups were dominated by *Christensenellaceae*. The B/C ratio might represent a metabolic trade‐off: *Blautia* enrichment could support butyrate‐mediated metabolic homeostasis and anti‐inflammatory effects, while reduced *Christensenellaceae* abundance may indicate a shift away from metabolic conservation strategies that become insufficient under physiological stress. This interpretation is consistent with existing research showing that *Blautia* has protective roles against metabolic syndrome, and although *Christensenellaceae* abundance is generally associated with leanness and metabolic health,^[^ ^71^, ^72^ ^]^ its reduction under high‐stress conditions could reflect an adaptive reallocation of resources toward more critical survival processes.

In parallel, we uncovered a distinct regulatory mechanism centered on L‐cysteine, another key mortality‐linked metabolite. We detected two colocalization signals in the jejunum and cerebellum, where the cis‐eQTL of the *PTER* and *ABCB1* genes were both associated with L‐cysteine levels. Recently studies have shown that the *PTER* gene is associated with body mass index (BMI), and that *PTER* knockout mice exhibit a complete loss of N‐acetyltaurine hydrolytic activity in tissues, resulting in a significant increase in systemic N‐acetyltaurine levels.^[^ ^73^ ^]^ This dual association may involve two distinct mechanisms: either *PTER* exerts pleiotropic effects on both metabolic pathways independently, or its primary impact on N‐acetyltaurine hydrolysis indirectly influences L‐cysteine levels, as the latter is the biosynthetic precursor of taurine and subsequently N‐acetyltaurine, linking the two pathways metabolically. The colocalization signals in these two organs may also suggest a potential relationship between the gut‐brain axis (GBA) and cysteine levels. L‐cysteine balances redox defense through two evolutionarily conserved pathways: GSH‐mediated Keap1/Nrf2 feedback inhibition^[^ ^52^, ^53^ ^]^ and direct thiol‐dependent reactivation of antioxidant enzymes,^[^ ^74^ ^]^ optimizing metabolic efficiency by coupling acute ROS neutralization with energy conservation.

Furthermore, the evolutionary conservation of metabolic pathways across species was demonstrated by the comparison of chicken and human metabolomes. The majority of human homologous metabolites associated with chicken mortality show causal associations with chronic disease. While the identification of 220 genes associated with mortality‐related biomarkers, including orthologs of human metabolic syndrome loci, highlights the potential for using poultry models to study human metabolic disorders.

We acknowledge several limitations in our study. First, the limited structural identification of MS features in chicken metabolomics highlights a critical challenge, restricting comparative analysis of conservation of metabolome biomarkers with well‐annotated human plasma metabolomes.^[^ ^75^, ^76^ ^]^ Second, our 16‐biomarker panel achieved exceptional discriminative capacity (AUC = 0.98 in discovery), yet its reduced external validity (AUC = 0.61 in validation) underscores environmental heterogeneity's role. This dichotomy highlights a critical limitation of metabolomics‐driven prediction models, in that biomarkers reflect real‐time physiological states, and their predictive power depends on standardized husbandry conditions. Third, the longitudinal profiling is warranted. This research only focused on single‐timepoint sampling (42 days of age), which may miss critical metabolic transitions during early growth phases. Lastly, the microbiome‐metabolite causality needs further validation, for which germ‐free models and fecal microbiota transplantation (FMT) experiments are needed to disentangle host‐microbe interactions, although our *MSRA*‐microbiota correlations suggest host genetics shapes microbial niches.

## 4 Conclusion

This study establishes comprehensive metabolic profiles for chicken mortality, identifies mortality‐associated biomarkers, and elucidates their genetic and metabolic bases. The integration of multi‐omics data not only provides a robust framework but also offers a model for studying metabolic trade‐offs across species, and can inform future research aimed at improving breeding strategies and understanding the complex interplay between metabolism and mortality. Our results show the value of metabolomics in understanding complex traits and provide insights for poultry breeding and human metabolic studies.

## 5 Experimental Section

### Study Design

A continuous homogeneous mortality mating model was implemented by tracking 42‐day mortality across full‐sibling families. In the G_0_ generation, broilers with divergent pedigree mortality (high mortality familie: sire, 8.5%; dam, 9.3% versus low mortality familie: sire, 1.5%; dam, 1.5%) were selectively mated to generate 181 G_1_ full‐sibling families. After adjusting mortality (as described in the Supporting Information), G_1_ progeny were stratified based on the corrected values into high‐mortality (H group, n = 115) and low‐mortality (L group, n  =  406) groups. To refine phenotypic resolution, G_2_ progeny (n = 102) were partitioned into gradient mortality subgroups (C, T1–T3) through iterative assortative mating. This multi‐generational design minimized inbreeding risks through avoiding 3‐generation inbreeding mating.

Genomic DNA from G_1_ samples was extracted using the phenol–chloroform method.^[^ ^77^ ^]^ Serum metabolomic profiling of G_1_ and G_2_ cohorts was performed in randomized batches to mitigate technical variability. For transcriptomics of heart, liver, and spleen, 20 broilers (10 from the H group and 10 from the L group) from the G_1_ cohort were euthanized via cervical dislocation under Animal Ethics Committee approval from the Institute of Animal Sciences (IAS), Chinese Academy of Agricultural Sciences (CAAS) (IAS2022‐37), and collected cardiac, hepatic, and splenic tissues immediately for RNA sequencing. From an independent validation cohort described in a previous study (n = 283), cecal contents were sampled to measure short‐chain fatty acids (SCFAs) with GC‐MS (Shimadzu QP‐2020 NX) and sequenced 16S rRNA genes (V3–V4 regions, Illumina NovaSeq 6000).^[^ ^45^ ^]^

### Metabolism Profiling

Serum metabolites were profiled using non‐targeted LC‐MS/MS, with relative abundances normalized using internal standards. Data preprocessing included batch correction and log_2_ transformation using the dplyr package (v3.5.0) in R. OPLS‐DA implemented in the ropls package (v1.6.2) identified mortality‐associated metabolites, with VIP scores ranking discriminatory features.

DAMs in serum were defined with stringent thresholds of |log_2_(fold change, H group / L group)| ≥ 0.58 and *P* < 0.05, which were computed via F‐statistics and Welch's *t*‐tests. The top 50 DAMs underwent hierarchical clustering (Euclidean distance and Ward's linkage method) and visualization using pheatmap (v4.3.2). Functional annotation integrated MetaboAnalyst 5.0 for metabolite set enrichment analysis (MSEA) and the OECloud platform (https://cloud.oebiotech.com/task/) for pathway mapping.^[^ ^78^ ^]^ Quality control metrics, including CV distributions and batch effect corrections, are detailed inSupporting Information. This analytical pipeline prioritized metabolites with both statistical significance and biological relevance, focusing on pathways mechanistically linked to mortality phenotypes.

### Metabolomic Genome‐Wide Association Studies

Genome‐wide mQTLs analysis was performed in G_1_ cohort using a linear mixed model (LMM) implemented in GEMMA (v0.98.4).^[^ ^79^ ^]^ Normalized metabolite intensities (log_2_(1+intensity)) were regressed against 590285 independent autosomal SNPs (pruned to –indep‐pairwise 25 5 0.2), adjusting for population structure via the top three genotype principal components (PCs). Genome‐wide significance was defined at *P* < 1.69 × 10^−6^ (Bonferroni correction threshold = 1/number of independent SNPs) to maximize the discovery of metabolite‐associated loci for comprehensive downstream characterization. Heritability (h^2^) was estimated via restricted maximum likelihood (REML) in GCTA (v1.93.2), incorporating genetic relatedness matrices and covariates.

### Biological Annotation for mQTLs

To explore potential associations between the mQTLs identified in this study and complex traits in chickens, the summary file “QTLdb_chickenGRCg6a.bed”was obtained and utilized from the Animal QTL Database (Animal QTLdb) (https://www.animalgenome.org/cgi‐bin/QTLdb/index) for annotation purposes.

To decode regulatory mechanisms, mQTLs were annotated against 15 chromatin states across 28 tissues using custom Perl scripts.^[^ ^80^ ^]^ Enrichment analysis employed Fisher's exact tests on 2 × 2 contingency tables, with Benjamini–Hochberg adjusted *P* quantifying chromatin state preference.

In addition, the mQTLs were integrated with the cis‐acting molecular QTLs (cis‐eQTL, cis‐exQTL, cis‐lncQTL, cis‐3′aQTL, and cis‐sQTL) identified in the Chicken GTEx to further explore their potential regulatory mechanisms.^[^ ^14^, ^15^ ^]^

### Genetic Contributions to DAMs Variation

To dissect the relative contributions of genetic regulatory variants (mQTLs) and Group‐level effects (H vs L) to DAMs variation, a hierarchical modeling framework was employed. First, competing linear models were formulated to test whether mortality‐associated differences in metabolite intensities were fully explained by mQTLs.

Reduced model (H0):

(1) $$\mathrm{DAMs}\;\mathrm{intensity}∼\mathrm{BW}+\mathrm{mQTLs}\;\mathrm{genotype}\;\left\{1..n\right\}$$

Full model (H1):

(2) $$\mathrm{DAMs}\;\mathrm{intensity}∼\mathrm{BW}+\mathrm{mQTLs}\;\mathrm{genotype}\;\left\{1..n\right\}+\mathrm{Group}$$

The significance of the Group effect was assessed via analysis of variance (ANOVA) using the stats R package (v0.3.0). Acceptance of H0 (*P* ≥ 0.05) indicated that biomarker differences between groups were fully attributable to mQTLs, whereas rejection (*P* < 0.05) suggested additional Group‐level influences.

To quantify variance components, a LMM using lme4 (v1.1–35.5) was applied:

(3) DAMsintensity∼BW+leadmQTLsgenotype+Group+ε

where *ε* represents residual variance. Variance proportions were calculated as: Proportion = REML estimate of random effect/Total variance. This approach partitioned contributions from Group effects, lead mQTL, and body weight to DAMs variation, thereby providing mechanistic insights into the interplay between genetic and environmental factors.

### Identification and Validation of Mortality‐Related Biomarkers

Two strategies were employed to identify mortality‐related biomarkers in Cohort 1. First, a LASSO regression with iterative 10‐fold cross‐validation (glmnet v4.1.3)^[^ ^81^ ^]^ was applied to select metabolites with stable associations with mortality, effectively balancing feature selection against overfitting through L1 regularization. Following feature selection via LASSO regression, an RF model was trained on 70% of the Cohort 1 samples and validated on the remaining 30% of Cohort 1, as well as on the entire Cohort 2, to assess the generalizable predictive utility of the identified metabolites. Second, a meta‐co‐expression network was constructed using the WGCNA R package (v1.70)^[^ ^82^ ^]^ to identify modules significantly associated with serum inflammatory cytokines, including T‐AOC, IL‐1β, and IFN‐γ. Principal component analysis (PCA) was applied to the metabolite intensity matrix within each module, the first principal component (module eigenvector, ME) was extracted to represent the module, resulting in an ME matrix.^[^ ^83^ ^]^ Here, metabolites linked to inflammatory cytokines and mortality were defined as metabolic biomarkers. The feature importance of metabolic biomarkers was conducted using the randomForest package (v 4.7–1.1) in R. Subsequently, an RF classifier was developed using the biomarkers identified via LASSO and WGCNA to predict class labels in Cohort 1. Hyperparameter optimization was conducted with the Hyperopt library (v0.2.7) in Python with a 7:3 training validation split. Receiver operating characteristic (ROC) curves were generated for the biomarkers, and the AUC was calculated using the pROC R package (v1.18.5). This integrative pipeline synergized dimension reduction (LASSO), network biology (WGCNA), and machine learning (RF) to overcome limitations inherent in single‐method approaches, as demonstrated in recent multi‐omics biomarker studies.^[^ ^84^ ^]^

### Cross‐Species Validation of Mortality‐Related Biomarkers

To systematically validate the conservation and efficacy of signature metabolites across evolutionary lineages and elucidate the mechanistic basis of broiler chicken mortality, a multi‐stage analytical workflow was employed. This approach specifically examined metabolites associated with acute pathological processes and aging‐related pathways to identify fundamental mortality drivers in poultry. The analysis commenced with Cohort 1, where the data were partitioned into training (80%) and testing (20%) subsets using a fixed random seed (set.seed(123)) to ensure reproducibility. The performance of six machine learning algorithms: logistic regression, decision trees, discriminant analysis, K‐nearest neighbors (KNN), naive Bayes, and bagged decision trees was assessed through 10‐fold cross‐validation. Model performance was evaluated based on accuracy, precision, recall, F1‐score, and AUC, with results visualized via multi‐model ROC curves and performance bar plots (Figure S22, Supporting Information).

Subsequent cross‐generational validation utilized a discriminant analysis model, identified as the optimal classifier during initial evaluation, which was trained exclusively on Cohort 1. This model was rigorously assessed against two distinct cohorts: the held‐out G_1_ test set (20% of Cohort 1 data) and the entirely independent Cohort 2. Performance was quantified using AUC and accuracy metrics, with ROC curves generated to evaluate robustness. To establish the cross‐species relevance of the signature metabolites, two streamlined metabolite panels were validated: a core set of three metabolites and an expanded panel containing the core three plus two cytokine‐associated metabolites. Both panels underwent parallel validation in chicken models (transferring from Cohort 1 training to Cohort 2 validation) and human models (Cohort 3, applying models trained on Verify 1 to Verify 2 cohorts). This design enabled direct assessment of the evolutionary conservation of the identified metabolites.

To investigate the fundamental mechanisms underlying broiler chicken mortality, literature‐derived metabolites associated with survival outcomes, and acute pathological processes were evaluted. These metabolites were subjected to identical cross‐species validation frameworks: models trained using these specific metabolite sets on the Cohort 1 (chicken) and Verify 1 (human) training data were rigorously tested on their respective hold‐out test sets and independent validation cohorts (Cohort 2 for chickens, Verify 2 for humans). To comprehensively characterize the combinatorial effects of metabolites, 3000 random metabolite combinations (from 1 to 32 metabolites for chickens and 1–16 metabolites for humans) were generated and evaluated using the established training/validation paradigm.

Metabolic markers associated with aging, acute pathological processes, and cytokine responses were primarily derived from published studies by Qiu et al,^[^ ^85^ ^]^ Sebastiani et al,^[^ ^86^ ^]^ Gong et al,^[^ ^87^ ^]^ Gao et al,^[^ ^88^ ^]^ Yang et al,^[^ ^89^ ^]^ McCann et al,^[^ ^90^ ^]^ and Nogal et al,^[^ ^91^ ^]^ with human‐specific metabolites obtained from Xu et al.^[^ ^92^ ^]^ A full list of these metabolic markers is provided in Table S4 (Supporting Information). All analyses were conducted in R (v4.3.1) using the following computational framework: model training and cross‐validation were implemented via the caret package, ROC analysis was performed with the pROC package, and data manipulation was executed using the dplyr package. Discriminant analysis served as the primary classifier throughout validation phases, with AUC as the principal performance metric. To ensure methodological reproducibility, random seeds were systematically fixed at all stochastic computational stages.

### Integration of mQTLs‐mol QTLs Regulatory Networks

To investigate shared genetic regulatory links between metabolic and transcriptional regulation, colocalization of mQTLs and molQTLs (cis‐eQTL, cis‐exQTL, cis‐lncQTL, cis‐3′aQTL, and cis‐sQTL) were analyzed from the Chicken GTEx with the coloc R package (v5.2.3).^[^ ^93^ ^]^ Employing a Bayesian framework, posterior probabilities for five hypotheses: 1) H0: no link to either phenotype, 2) H1: link to only the first phenotype, 3) H2: link to only the second phenotype, 4) H3: links to both phenotypes from separate signals, and 5) H4: links to both phenotypes through shared signals were calculated. Colocalization confidence were rated as strong (PH4 ≥ 0.8) or moderate (0.5 < PH4 < 0.8) using recent standard thresholds.^[^ ^94^ ^]^

### Precision Fine‐Mapping of Causal Variants

Causal variants underlying biomarker‐associated mQTLs were resolved through a dual‐strategy framework: 1) FINEMAP (v1.4)^[^ ^95^ ^]^: Leveraged shotgun stochastic search to identify causal configurations within linkage disequilibrium (LD)‐defined regions (r^2^ > 0.8, calculated via PLINK v1.90). This approach computes Bayes factors for variant inclusion while accounting for allelic heterogeneity. 2) Sum of Single Effects (SuSiE)^[^ ^96^ ^]^: Implemented via susieR (v0.12.35) using iterative Bayesian stepwise selection to decompose genetic signals into up to five causal variants per locus.

Model convergence was assessed through diagnostic plots of posterior inclusion probabilities (PIP) and credible set sizes. Variants with PIP > 0.8 were classified as high‐confidence causal and were supported by both Bayesian and frequentist criteria.^[^ ^97^ ^]^ This integrative approach reconciled complementary strengths of stochastic search (FINEMAP) and sparse effect modeling (SuSiE), addressing limitations of single‐method analyses in polygenic architectures.

### Vitro Validation of Antioxidant Mechanisms

HD11 chicken macrophage‐like cells were maintained in RPMI‐1640 medium (10% FBS, 1% penicillin‐streptomycin) at 37 °C under 5% CO_2_. To establish oxidative stress conditions, cells were exposed to 3% H_2_O_2_ (736.4 µm, 2 h) (Figure S34A, Supporting Information) following preliminary dose‐response optimization. Pharmacological interventions included: 1) Nrf2 inhibition: ML385 (2.95 µm, 48 h; MedChemExpress, USA) (Figure S34B, Supporting Information),^[^ ^98^ ^]^ 2) Nrf2 activation: IR‐61 (17.9 µm, 48 h; Shaanxi New Research Bioscience, China) (Figure S34C, Supporting Information),^[^ ^99^ ^]^ 3) Antioxidant treatment: L‐cysteine (68.33 µm, 48 h; MedChemExpress, USA) (Figure S34D, Supporting Information).

### Cell Viability and Oxidative Stress Profiling

Viability was quantified via cell counting kit‐8 (CCK‐8) assay (Beyotime, China). Cells (8000 per well in 96‐well plates) were incubated with treatments, followed by 2 h CCK‐8 exposure. Absorbance was measured at 450 nm using an RT 6000 microplate reader (Rayto, China).

### Protein Expression Analysis

Treated cells were lysed in radio immunoprecipitation assay (RIPA) buffer with protease inhibitors and centrifuged them at 14 000 rpm for 20 min at 4 °C. 30 µg of protein per lane were separated using sodium dodecyl sulfate‐polyacrylamide gel electrophoresis (SDS‐PAGE), transferred them to PVDF membranes (Millipore, USA), and detected Nrf2, NQO1, HO‐1, and β‐actin with antibodies at a 1:1000 dilution. After incubating with secondary antibodies for 1 h at room temperature, the blots were visualized with ECL and captured images using the Tanon Gel Image System (v4.00, China).

### Oxidative Stress Biomarker Quantification

Antioxidant capacity was assessed using commercial kits (Nanjing Jiancheng Bioengineering, China): 1) Enzymatic activity: CAT (EC1.11.1.6), GPx (EC1.11.1.9), SOD (EC1.15.1.1), 2) Lipid peroxidation: MDA levels via thiobarbituric acid assay. All experiments included three biological replicates and vehicle controls.

### Statistical Analysis

Data were presented as the mean ± standard error of the mean. For comparisons between two groups, a two‐sided Wilcoxon rank‐sum test (Mann‐Whitney U test) was used by default, with *P* < 0.05 considered statistically significant. However, for comparisons of MAF, phastCons scores, and chCADD scores between mQTL and non‐mQTL variants, Student's *t*‐test was applied instead, owing to the large sample sizes. Statistical significance in figures was indicated as follows: *P* < 0.05 (^*^), *P* < 0.01 (^**^), *P* < 0.001 (^***^), and *P* < 0.0001 (^****^). The specific statistical test applied and the sample size (n) for each analysis were explicitly stated in the corresponding figure legends. The associations with mortality and microbial abundance were evaluated using Kendall's tau coefficient, while the relationship between gene expression and metabolite intensity was assessed using Spearman's correlation. For all other correlations, unless otherwise stated, Pearson's correlation was applied. Statistical significance of all correlations was tested, and results were visualized using the corrplot R package (v0.95). Unless otherwise specified, visualizations were conducted using the ggplot2 R package (v3.5.0). For all boxplots, horizontal lines inside the boxes show the medians. Box bounds show the lower quartile (Q1, the 25th percentile) and the upper quartile (Q3, the 75th percentile).

## Conflict of Interest

The authors declare no conflict of interest.

## Supporting information



Supporting Information

Supplemental Table 1‐7

## Data Availability

The metabolomics dataset generated in this study has been submitted to the China National Center for Bioinformation (CNCB) OMIX database under the accession numbers OMIX009728 and OMIX009730, and the BioProject numbers PRJCA038365 and PRJCA038367. The whole‐genome resequencing data are available in the GSA database of the China National Genomics Data Center (NGDC) under the accession number CRA024555 and the BioProject accession number PRJCA038233. The RNA‐seq data are available in the GSA database of the NGDC under the accession number CRA024448 and the BioProject number PRJCA038233. Furthermore, the data that support the findings of this study are available from the corresponding author upon reasonable request.
