# Supplementary material for: Integrative Omics Defines Metabolic Biomarkers and Genetic Regulatory Mechanisms of Mortality Risk
Source: Adv Sci (Weinh). 2025 Nov 18;13(3):e14464. doi: 10.1002/advs.202514464 (PMC12806298; doi:10.1002/advs.202514464)
Supplement: Supplementary file 1 — Supporting Information [file ADVS-13-e14464-s001.docx]

Supporting Information

**Integrative omics defines metabolic biomarkers and genetic regulatory mechanisms of mortality risk**

*Peihao Liu, Bingxing An, Jumei Zheng, Qiao Wang, Zhirui Yang, Zhengda Li, Dawei Liu, Fan Ying, Jie Wen, Lingzhao Fang^*^, and Guiping Zhao^*^*

**Table of Contents**

**1.** **Supplementary Methods**

**1.1. Animal Care and Handling**

**1.2. Calculation of Adjusted Mortality**

**1.3. Serum Metabolomic Profiling by LC-MS/MS**

**1.4. Quantification of Serum Inflammatory Cytokines**

**1.5. Genotyping and Quality Control**

**1.6. Calculation of Genetic Variance Explained for Metabolite Traits**

**1.7. Functional Annotation with ChCADD**

**1.8. Evolutionary Conservation Analysis with PhastCons**

**1.9. Calculation of Metabolite Fold Change**

**1.10. RNA-seq Data Processing and Bioinformatics Analysis**

**1.11. Differentially Expressed Genes Analysis**

**1.12. Cecal Microbiome Profiling and Analysis**

**1.13. Dual-Luciferase Reporter Gene Assay**

**1.14. Cross-Species Comparative Analysis of Metabolic Regulation**

**2. Supplementary Figures**

**3. Supplementary Tables**

**4. Supplementary References**

**1.** **Supplementary Methods**

**1****.1. Animal Care and Handling**

The experimental cohort comprised purebred white-feathered broilers (Xinguang Agricultural and Animal Industrials Co., Ltd., Mile, China), maintained under standardized environmental conditions (20-25 °C, 60-65 % relative humidity) in three-tier cage systems (1,250 × 1,000 × 600 mm). Both Cohort 1 (discovery cohort) and Cohort 2 (internal validation cohort) were reared from 0 to 42 days of age in the same facility under identical environmental conditions, feeding regimens, and husbandry practices. All birds were provided ad libitum access to a corn-soybean meal-based diet (composition detailed in Table S5, Supporting Information), with longitudinal phenotypic data (body weight, body weight gain, and mortality rates were recorded at 28 and 42 days of age.) systematically recorded.

For serum isolation, whole blood samples were collected from the wing vein into vacuum blood collection tubes, followed by immediate centrifugation (3,000 × g, 10 min, 4°C). Serum aliquots were flash-frozen in liquid nitrogen and stored at -80°C to preserve metabolic integrity until analysis.

**1.2. Calculation of Adjusted Mortality**

Under modern intensive poultry production systems, overall mortality rates are typically low. However, even modest differences in flock size can introduce substantial bias when comparing mortality rates across different lines. To correct for this size-related confounding effect, line-specific mortality was adjusted using the following formula:

$$adjusting mortality=\frac{crude mortality\times total standard mortality}{total expect mortality} （1）$$

$$crude mortality=\frac{Number of deceased individuals in the family}{Number of individuals in the family} （2）$$

$$total standard mortality=\frac{Average number of deaths in parental families}{Number of parental individuals} （3）$$

$$total expect mortality=\frac{Average number of deaths in parental families}{Average number of individuals in both parents' families}\times crude mortality （4）$$

**1.3.** **Serum Metabolomic Profiling by LC-MS/MS**

The sample preparation procedure of LC-MS/MS analysis was as follows: Serum samples stored at -80°C were thawed at room temperature. Next, 80 μL of each sample was transferred into a 1.5 mL Eppendorf tube containing 240 μL of a methanol:acetonitrile mixture (2:1, v/v) spiked with L-2-chlorophenylalanine (2 μg/mL). The mixtures were vortexed for 1 minute and subjected to ultrasonic extraction in an ice-water bath for 10 minutes. The samples were then stored at -40°C for 30 minutes and centrifuged at 12,000 rpm for 20 minutes at 4°C. After centrifugation, 150 μL of the supernatant was collected using crystal syringes, filtered through 0.22 μm microfilters, and transferred into LC vials. These vials were stored at -80°C until LC-MS/MS analysis.

Quality control (QC) samples were prepared by pooling equal aliquots from all serum samples to form a composite reference sample, which was used to monitor analytical reproducibility throughout the sequence. The high correlation (r = 0.99; Figure S36, Supporting Information) among QC samples confirmed stable instrument performance and data reliability. To further ensure data reliability, we employed a panel of four carefully selected internal standards: [²H₄]-succinic acid, L-2-chlorophenylalanine, D-luciferin free acid, and [²H₄]-cholic acid strategically chosen to represent diverse chemical classes including organic acids, amino acid analogues, and bile acids. The relative standard deviations (RSDs) of these internal standards were all below 30%, and retention time shifts were constrained within ±18 seconds, collectively attesting to the stability of the analytical process and reproducibility of the chromatographic system.

All samples were analyzed using an ACQUITY UPLC I-Class Plus system (Waters Corporation, Milford, USA) coupled with a Q-Exactive mass spectrometer equipped with a heated electrospray ionization (ESI) source (Thermo Fisher Scientific, Waltham, MA, USA). Chromatographic separation was performed on an ACQUITY UPLC HSS T3 column (1.8 μm, 2.1 × 100 mm) maintained at 45°C, using a mobile phase consisting of water with 0.1% formic acid and acetonitrile delivered at a flow rate of 0.35 mL/min. The injection volume was 2 μL, and samples were stored at 10°C in the autosampler during analysis. Mass spectrometry signals were acquired in both positive and negative ionization modes. Detailed acquisition parameters and chromatographic program settings are provided in Table S6 (Supporting Information).

The original data were processed using Progenesis QI software (v2.3) (Nonlinear Dynamics, Newcastle, UK) for baseline filtering, peak detection, integration, retention time correction, peak alignment, and normalization. Key parameters included a precursor tolerance of 5 ppm, a product tolerance of 10 ppm, and a product ion threshold of 5%. Compound identification was based on accurate mass-to-charge ratios (m/z), secondary fragment analysis, and isotopic distribution, utilizing databases such as Metlin,^[1]^ LIPID Maps (v2.3),^[2]^ the Human Metabolome Database (HMDB).^[3]^ The extracted data were refined by excluding peaks with missing values (ion intensity = 0) in more than 50% of samples within any experimental group. Missing values were replaced with half of the minimum detected value. Compounds were then screened based on qualitative scores, with scores below 36 (out of 60) considered unreliable and excluded from further analysis. Finally, data from positive and negative ion modes were merged to create a comprehensive data matrix.

**1.4. Quantification of Serum Inflammatory Cytokines**

To compare the inflammatory and immune responses between the divergent H and L groups, a subset of 79 serum samples from Cohort 1 (randomly selected from within each line; H:L = 23:56) was analyzed. Measurements were performed using commercial enzyme-linked immunosorbent assay (ELISA) kits (Nanjing Jiancheng Bioengineering Institute, China) according to the manufacturer’s instructions.^[4]^ The measured analytes included the inflammatory cytokines interleukin-1β (IL-1β) and interferon-γ (IFN-γ), as well as the total antioxidant capacity (T-AOC) as a global measure of oxidative stress.

**1.5.** **Genotyping and Quality Control**

Paired-end sequencing libraries were constructed from genomic DNA with an insert size of 300-500 bp. Sequencing was conducted on the DNBSEQ-T7 platform by by Higentec Co., Ltd in Changsha, China, achieving an average coverage of 10×. Quality control of the raw sequencing data was performed using FASTP (v0.23.4).^[5]^

Clean reads were aligned to the chicken reference genome (GRCg6a/galGal6, https://ftp.ncbi.nlm.nih.gov/genomes/all/GCF/000/002/315/GCF_000002315.6_GRCg6a/) using Sentieon software (v202112.05), and variants were called to generate gVCF files.^[6]^ SNPs were subsequently filtered using Sentieon (v202112.05) with the following expression: QualByDepth (QD) < 2.0 || FisherStrand (FS) > 60.0 || MappingQuality (MQ) < 35.0 || MQRankSum < -12.5 || ReadPosRankSum < -8.0 .

Further quality control was conducted using PLINK (v1.90)^[7]^ with the following filters applied: minor allele frequency (MAF) ≥ 5%, variant genotyping rate ≥ 90%, individual genotyping rate ≥ 90%, and exclusion due to significant deviation from Hardy-Weinberg equilibrium (*P* < 10⁻⁵). After applying these filters, 9,053,630 autosomal variants were retained for the Cohort 1 of broilers (Figure S7, Supporting Information). The linkage disequilibrium (LD) decay analysis was performed using PopLDdecay software (v3.43).^[8]^

**1.6. Calculation of Genetic Variance Explained for Metabolite Traits**

The phenotypic variance explained (PVE) by metabolite quantitative trait locus (mQTL) was calculated according to the method proposed by Shim et al.^[9]^

$$PVE=\frac{2{\overset{\wedge}{\beta}}^{2}MAF(1-MAF)}{{2{\overset{\wedge}{\beta}}^{2}MAF\left( 1-MAF \right)+(se(\overset{\wedge}{\beta}))}^{2}2NMAF(1-MAF)} （5）$$

where $\overset{\wedge}{\beta}$is the effect size of the SNP marker, MAF is the minor allele frequency of the SNP markers, se ($\overset{\wedge}{\beta}$) is the standard error of the effect size for the SNP marker, and N is the sample size.

**1.7. Functional Annotation with ChCADD**

The chicken combined annotation-dependent depletion (chCADD) score was used to identify specific subregions of potentially higher functional importance within each conserved non-coding element (CNE), assuming that high-scoring mQTLs indicate such regions.^[10]^ The chCADD scores were available from OSFHOME (https://osf.io/8gdk9/).

**1.8.** **Evolutionary Conservation Analysis with PhastCons**

To understand the evolutionary conservation of mQTLs, we assessed their sequence conservation using phastCons scores derived from 77 vertebrates. The phastCons scores for chicken autosomal chromosomes (1-28) were downloaded from the UCSC database, and the corresponding reference sequences were obtained from the Ensembl database (https://asia.ensembl.org/). The conservation analysis was performed using the SnpSift tool integrated within SnpEff (v5.0).^[11, 12]^

**1.9.** **Calculation of Metabolite Fold Change**

In this study, we computed the metabolite fold change (mFC) to quantify the influence of mQTLs on metabolite abundance. This biologically interpretable measure is defined as the base-2 logarithm of the ratio of median metabolite abundances between alternative (mutant-type, BB) and reference (wild-type, AA) homozygous genotypes, providing a standardized and robust effect size metric for mQTL mapping. This median-based approach provides a standardized measure of both the direction and magnitude of the genetic effect while offering inherent resistance to outliers and non-normally distributed data. For each metabolite–genotype pair, samples were stratified by allele dosage (0, 1, or 2 representing AA, AB, and BB genotypes).

When both AA and BB groups had non-missing observations, the median fold change was defined as:

$$\mathrm{mFC}=\log_{2} \left( \frac{\mathrm{median}(\mathrm{BB})}{\mathrm{median}(\mathrm{AA})} \right) （6）$$

If the BB group was absent but AB was available, an approximation was applied:

$$\mathrm{mFC}=\log_{2} \left( \frac{2\times\mathrm{median}(\mathrm{AB})}{\mathrm{median}(\mathrm{AA})}-1 \right) （7）$$

In these expressions, median(AA), median(AB), and median(BB) represent the medians of the covariate-adjusted metabolite phenotypes within the reference homozygous, heterozygous, and alternative homozygous groups, respectively.The above calculations were implemented in Perl (v5.16.3).

Prior to mFC calculation, metabolite intensity values were adjusted for technical and biological confounders using linear regression. For each metabolite j, we fitted the model:

$$y_{ij}=\beta_{0}+\beta_{1}\times\mathrm{Group}_{i}+\beta_{2}\times\mathrm{BW}+\beta_{3}\times{\mathrm{PC}1}_{i}+\beta_{4}\times{PC2}_{i}+\beta_{5}\times{\mathrm{PC}3}_{i}+\epsilon_{ij} （8）$$

where $y_{ij}$is the intensity of metabolite j in sample i. The covariates adjust for experimental group (Group, categorical variable with multiple levels), body weight at day 42 (BW, continuous), and population stratification (the first three genetic principal components PC1–PC3, continuous). Categorical covariates exhibiting only one level for a given metabolite were excluded to prevent model singularities. The residuals ($\epsilon_{ij}$​) from this model were extracted as the adjusted metabolite abundances for all subsequent mFC calculations. This covariate adjustment process was implemented in R (v4.4.1).

**1.10. RNA-seq Data Processing and Bioinformatics Analysis**

Total RNA was extracted using TRIzol Reagent (Invitrogen, Carlsbad, CA, USA), and RNA integrity was assessed with an Agilent 2100 Bioanalyzer (Agilent Technologies, Santa Clara, CA, USA). All RNA samples (20 per tissue type across three tissues, 60 samples in total) met quality standards for cDNA library construction and were processed using the VAHTS Universal V6 RNA-Seq Library Prep Kit (Vazyme, Nanjing, China). Libraries were sequenced on the Illumina NovaSeq 6000 platform (Illumina, San Diego, CA, USA), producing 150 bp paired-end reads and approximately 45 million raw reads per sample (Table S7, Supporting Information). Raw reads in FASTQ format were processed using FASTP to remove low-quality sequences, yielding clean reads for downstream analyses. These reads were aligned the reference genome (GRCg6a/galGal6) using HISAT2 (v2.1.0)^[13]^ and gene-level read counts were quantified using HTSeq-count (v0.11.2).^[14]^

**1.11. Differentially Expressed Genes Analysis**

Differentially expressed genes (DEGs) between the H and L groups were analyzed using the DESeq2 R package (v1.16.1). The Benjamini-Hochberg method was employed to adjust *P* (p-adjust) for multiple testing. DEGs were defined by a p-adjust < 0.05 and a fold-change (FC) thresholds of >1.2 or <0.83. Gene functional enrichment analysis was performed using the R package clusterProfiler (v4.12.1) To ensure compatibility with downstream annotation resources, Ensembl gene IDs were first converted to NCBI Entrez Gene IDs using the Omicshare platform (https://www.omicshare.com). Enrichment analysis was subsequently carried out using the Gallus gallus OrgDb annotation package org.Gg.eg.db.

**1.12.** **Cecal Microbiome Profiling and Analysis**

Based on the study by He et al,^[15]^ we selected broilers with extremely high (HB, n = 50, mean = 0.41 μg/mg) and low (LB, n = 50, mean = 0.06 μg/mg) levels of cecal butyric acid to compare microbial composition. Relative abundances at the phylum and genus levels were statistically compared between the groups.

Community diversity was assessed using three indices: the Simpson index (species dominance and evenness), the Shannon index (community diversity), and Observed Richness (number of observed taxa). Beta diversity (inter-sample variation, Bray-Curtis dissimilarity) was evaluated via principal coordinate analysis (PCoA) to visualize sample clustering and highlight intergroup differences. The Simpson index, Shannon index, Richness index, and beta diversity were calculated using the vegan R package (v2.5-7).

Statistical analysis of metagenomic profiles (STAMP) was performed to identify genera with significant differences between the groups. Linear discriminant analysis (LDA) effect size (LEfSe) was applied to identify biomarker taxa with differential abundance between groups using the microeco R package (v1.10.0). Kendall’s tau correlation coefficients between butyric acid levels and microbial genera were calculated using the ggpubr R package (v0.6.0).

To further investigate the genetic basis underlying cecal butyric acid levels and microbial abundance, we performed a genome-wide association study (GWAS) using a linear mixed model (LMM) implemented in GEMMA (v0.98.4) on 283 individuals. Independent genetic loci associated with microbial abundance and butyrate levels were selected as instrumental variables based on a significance threshold of *P* < 1×10^-5^, and clumping for linkage disequilibrium was performed using the parameters r² < 0.1 and a window size of 50 kb.We then applied Mendelian randomization (MR) analysis using the TwoSampleMR R package (v0.6.9) to infer potential causal relationships among cecal microbiota, cecal butyric acid levels, and systemic serum butyrate levels.

**1.13. Dual-Luciferase Reporter Gene Assay**

DNA fragments containing the rs318007359(A/T) and rs732044877(A/G) alleles were amplified by artificial synthesis and site-directed mutagenesis. These fragments were then cloned into the pGL4.18 firefly luciferase expression vector to generate *PTER* and *MSRA* promoter reporter plasmids carrying the respective alleles. 293T cells were seeded into 48-well plates for dual-luciferase reporter gene assays. Cells were cultured in RPMI-1640 medium supplemented with 10% fetal bovine serum (FBS) and maintained in a humidified incubator at 37°C with 5% CO_2_. After transfection with the reporter constructs for 48 hours, luciferase activity was measured using the Dual-Luciferase Reporter System (Promega Corporation, Madison, WI, USA). Four independent transfection experiments were conducted for each plasmid construct.

**1.14. Cross-Species Comparative Analysis of Metabolic Regulation**

To investigate the evolutionary conservation of metabolic regulation between broiler and human, we conducted a series comparative analyses focused on mortality associated metabolites and their regulatory genes. First, we integrated the results of a published MR analysis,^[16]^ along with those from a metabolomic analysis related to human lifespan,^[17]^ to investigate potential associations between signature metabolites associated with mortality in white feathered broilers and human disease. Subsequently, transcriptome-wide association study (TWAS) and genomic datasets from human metabolic syndrome studies were utilized to evaluate the genetic contributions of these metabolite associated genes to complex diseases.^[18]^ Additionally, we compared the genes associated with the mortality-related signature metabolites identified in this study to those linked to metabolic syndrome (MetS) to investigate the conservation of metabolic regulatory mechanisms between chickens and humans.^[19]^ Furthermore, GO functional annotation enrichment analyses were performed using the clusterProfiler R package (v4.12.1), identifying evolutionarily conserved metabolic regulatory networks.

**2. Supplementary Figures**

Figure S1. Differential analysis of mortality in experimental cohorts.

Figure S2. Genetic structure of H and L groups in Cohort 1.

Figure S3. Comparative analysis of cytokine levels and growth performance in Cohort 1.

Figure S4. Comprehensive metabolic characterization of Cohort 1.

Figure S5. Heatmap of the top 50 significantly differentially accumulated metabolites between H and L groups in Cohort 1.

Figure S6. Enrichment analysis of metabolites.

Figure S7. Genome-wide SNP distributions in Cohort 1.

Figure S8. Functional and statistical profiling of mQTLs.

Figure S9. Metabolite associated pleiotropic mQTLs network on chromosome 1.

Figure S10. Functional enrichment analysis of pleiotropic mQTLs associated genes.

Figure S11. Number of complex trait-associated mQTLs across metabolite super classes.

Figure S12. Colocalization analysis of mQTLs and molecular QTLs from the Chicken GTEx.

Figure S13. Hepatic transcriptomic profiling of genes shared between metabolite and molecular QTLs.

Figure S14. Pearson correlation of MAF for metabolic biomarker-associated SNPs between H and L groups.

Figure S15. Heritability analysis of metabolic biomarkers.

Figure S16. Metabolic canalization driven by genetic-metabolic trade-offs under differential mortality.

Figure S17. Assessment of group-level influences on metabolites levels.

Figure S18. LASSO regression for identification of key metabolites associated with mortality.

Figure S19. Network construction and module identification.

Figure S20. Characterization of metabolic biomarkers.

Figure S21. Correlation analysis of metabolic biomarkers.

Figure S22. Model evaluation for mortality prediction.

Figure S23. Conserved metabolic regulation between chickens and humans.

Figure S24. Functional enrichment analysis of metabolic biomarkers associated genes.

Figure S25. Causal effects of metabolic biomarkers on complex diseases in the Han Chinese population.

Figure S26. Abundance of butyrate and L-cysteine in H and L groups.

Figure S27. GWAS for butyrate and L-cysteine.

Figure S28. GWAS for cecal butyrate and related microbial taxa.

Figure S29. Causal effect of cecal butyrate on serum butyrate levels assessed by Mendelian randomization analysis.

Figure S30. Butyrate-associated gut microbiota profiling.

Figure S31. Correlation between butyrate and microbial features.

Figure S32. Genomic context of the L-cysteine associated loci on chromosome 2.

Figure S33. Cross-tissue correlation between gene expression and metabolites.

Figure S34. Determination of half maximal inhibitory concentration for H_2_O_2_, ML385, IR-61, and L-cysteine.

Figure S35. Protein expression of Nrf2 pathway components across treatments.

Figure S36. Correlation matrix illustrating the high reproducibility among 20 quality control samples.

**Figure S1.** Differential analysis of mortality in experimental cohorts. A) Mortality between H and L groups in G_0_ cohort. Significance was determined by Wilcoxon test, **** *P* < 0.0001. B) Mortality comparison among all groups in Cohort 2. Significance was determined by Wilcoxon test, **** *P* < 0.0001.

**Figure S2.** Genetic structure of H and L groups in Cohort 1. A) Linkage disequilibrium (LD) decay. B) Principal component analysis (PCA).

**Figure S3.** Comparative analysis of cytokine levels and growth performance in Cohort 1. A) Serum levels of T-AOC, IL-1β, and IFN-γ differed significantly between H and L groups. Significance was determined by Wilcoxon test, NS *P* > 0.05, **** *P* < 0.0001. B) Weekly weight gain during the 5th and 6th weeks. Significance was determined by Wilcoxon test, NS *P* > 0.05, **** *P* < 0.0001.

**Figure S4.** Comprehensive metabolic characterization of Cohort 1. A-B) Chromatograms of negative and positive ion modes from untargeted serum metabolomics analysis. C) Comparison of the coefficient of variation (CV) between H and L groups. Significance was determined by Wilcoxon test, * *P* < 0.05. D) Distribution of CV values for all metabolites in H and L groups.

**Figure S5.** Heatmap of the top 50 significantly differentially accumulated metabolites between H and L groups in Cohort 1. The content data for each metabolite were normalized.

**Figure S6.** Enrichment analysis of metabolites. A) Kyoto Encyclopedia of Genes and Genomes (KEGG) pathways enriched by metabolites. B) Metabolite set enrichment analysis (MSEA) results for all metabolites.

**Figure S7.** Genome-wide SNP distributions in Cohort 1. A) Distribution of minor allele frequency (MAF) across chromosomes 1 to 28. B) Number of high-quality SNPs per linkage disequilibrium (LD) block. The median number of SNPs per LD block is 3 (distribution of LD block size is shown in the inset). C) Distribution of SNP number across per chromosome.

**Figure S8.** Functional and statistical profiling of mQTLs. A) Distribution of minor allele frequency (MAF) for mQTL and non-mQTL. Significant differences were determined by t-test, *** *P* < 0.001. B) Distribution of phastCons conservation scores for mQTL and non-mQTL. Significant differences were determined by t-test, ** *P* < 0.01. C) Distribution of distances to transcription start sites for mQTL and non-mQTL. Significant differences were determined by t-test, *** *P* < 0.001. D) Distribution of all SNPs (9,053,630) and mQTLs across eight genomic features. E) Distribution of chCADD scores for mQTLs. F) Enrichment of mQTL in 15 chromatin states across 23 chicken tissues. G) Genome-wide distribution of metabolite fold change (mFC) values for mQTLs.

**Figure S9.** Metabolite associated pleiotropic mQTLs network on chromosome 1. Circular nodes represent mQTLs, colored by their chromosomal region. Square nodes represent metabolites, colored by their metabolite super class.

**Figure S10.** Functional enrichment analysis of pleiotropic mQTLs associated genes. A) Top 15 enriched KEGG pathways for pleiotropic mQTLs genes. B) Top 15 enriched GO terms for pleiotropic mQTLs genes.

**Figure S11.** Number of complex trait-associated mQTLs across metabolite super classes. A-I) The number of mQTLs for each super class: A) Unclassified, B) Others, C) Phenylpropanoids and polyketides, D) Organoheterocyclic compounds, E) Organic oxygen compounds, F) Organic acids and derivatives, G) Nucleosides, nucleotides, and analogues, H) Lipids and lipid-like molecules, I) Benzenoids.

**Figure S12.** Colocalization analysis of mQTLs and molecular QTLs from the Chicken GTEx. A) Distribution of the posterior probability for colocalization (PH4) between mQTLs and five types of molQTLs. B) Number of colocalization events (PH4 > 0.5) between nine metabolite classes and five molQTL types.

**Figure S13.** Hepatic transcriptomic profiling of genes shared between metabolite and molecular QTLs. A) Differential expression analysis of protein-coding genes. B) Differential expression of genes implicated in both mQTL and molQTL signals.

**Figure S14.** Pearson correlation of MAF for metabolic biomarker-associated SNPs between H and L groups.

**Figure S15.** Heritability analysis of metabolic biomarkers. A) Distribution of heritability estimates in the L group calculated using different sample subsets (n = 80, 180, 280, and the full cohort). Significance was determined by Wilcoxon test, ns *P* > 0.05. B) Comparison of heritability distributions between the H and L groups. Significance was determined by Wilcoxon test, ns *P* > 0.05. C) Heritability estimates for metabolites across different QTL sets. Significance was determined by Wilcoxon test, **** *P* < 0.0001. D) Distribution of genetic variance (V(G)) explained by different QTL sets. Significance was determined by Wilcoxon test, ** *P* < 0.01.

**Figure S16.** Metabolic canalization driven by genetic-metabolic trade-offs under differential mortality. A) Proportion of phenotypic variance in DAMs explained by group effect, top mQTL, and body weight (BW). Significance was determined by Wilcoxon test. NS *P* > 0.05, **** *P* < 0.0001. B) Comparison of L-cysteine intensity among individuals with different genotypes at the rs317441580 locus between the H and L group in Cohort 1.

**Figure S17.** Assessment of group-level influences on metabolites levels. A) Comparison of F-statistics from the reduced and full models. B) Correlation of *P* obtained from Student’s t-test and ANOVA.

**Figure S18.** LASSO regression for identification of key metabolites associated with mortality. A) LASSO coefficient profiles of 92 candidate key metabolites. B) Tuning parameter selection via 10-fold cross-validation with minimum criteria in the LASSO model.

**Figure S19.** Network construction and module identification. A) Scale independence (left) and mean connectivity (right) for choosing the soft threshold in WGCNA. B) Cluster dendrogram and topological overlap heatmap of metabolites. C) Dendrogram showing identified metabolite modules. D) Distribution of correlation coefficients with IFN-γ across metabolite modules. E) Association between metabolites in the brown module and IFN-γ levels. F) Correlation between IFN-γ and 2,6,6-Trimethyl-2-cyclohexene-1,4-dione.

**Figure S20.** Characterization of metabolic biomarkers. A) Overlap between key metabolites identified by LASSO regression and the WGCNA brown module. B) Association between metabolic biomarker levels and mortality risk. C) Predictive performance (AUC) of biomarker sets: WGCNA brown module (yellow), LASSO metabolites (blue), and their intersection (red). D) Distribution of coefficient of variation (CV), fold change (FC), and variable importance in projection (VIP) scores across super classes for all biomarkers. Red dots represent mean values in Cohort 1.

**Figure S21.** Correlation analysis of metabolic biomarkers. A) Correlation between hexyl glucoside and butyrate intensity. B) Correlation analysis of the metabolic markers. C) Correlation between hexyl glucoside and L-cysteine intensity.

**Figure S22.** Model evaluation for mortality prediction. A) Receiver operating characteristic (ROC) curves of six classifiers in Cohort 1. B) Precision, recall, and F1-score for the six candidate models.

**Figure S23.** Conserved metabolic regulation between chickens and humans. A) Venn diagram of metabolic biomarker-associated genes and genes linked to metabolic syndrome (MetS). B) Association between metabolic biomarker-associated genes and mortality risk in Cohort 1.

**Figure S24.** Functional enrichment analysis of metabolic biomarkers associated genes. A) Top 15 enriched KEGG pathways for metabolic biomarkers associated genes. B) Top 15 enriched GO terms for metabolic biomarkers associated genes.

**Figure S25.** Causal effects of metabolic biomarkers on complex diseases in the Han Chinese population. A-E) Mendelian randomization (MR) analysis revealing causal effects of A) cysteine, B) S-allyl-L-cysteine, C) S-glutathionyl-L-cysteine, D) LysoPC(0:0/18:2), and E) LysoPC(0:0/18:3) on chronic diseases.

**Figure S26.** Abundance of butyrate and L-cysteine in H and L groups. A) Intensity distribution of butyrate across groups in Cohort 1. Significance was determined by Wilcoxon test, *** *P* < 0.001. B) Intensity distribution of butyrate across groups in Cohort 2. C) Intensity distribution of L-cysteine among different groups in Cohort 1. Significance was determined by Wilcoxon test, *** *P* < 0.001. D) Intensity distribution of L-cysteine among different groups in Cohort 2.

**Figure S27.** GWAS for butyrate and L-cysteine. A) Manhattan plot for butyrate associated loci. B) Quantile-quantile (Q-Q) plot for butyrate GWAS. C) Manhattan plot for L-cysteine associated loci. D) Q-Q plot for L-cysteine GWAS.

**Figure S28.** GWAS for cecal butyrate and related microbial taxa. A-B) Manhattan and Q-Q plot for the genome-wide association analysis of cecal butyrate levels. C-D) Manhattan and Q-Q plot for the genome-wide association analysis of cecal *Blautia* abundance. E-F) Manhattan and Q-Q plot for the genome-wide association analysis of cecal *Christensenellaceae_R-7_group* abundance. G-H) Manhattan and Q-Q plot for the genome-wide association analysis of the *Blautia*/*Christensenellaceae_R-7_group* (B/C) ratio.

**Figure S29.** Causal effect of cecal butyrate on serum butyrate levels assessed by Mendelian randomization analysis.

**Figure S30.** Butyrate-associated gut microbiota profiling. A) PCoA based on weighted UniFrac distance for the entire dataset. B) Comparison of α-diversity indices (Richness, Simpson, Shannon) between HB and LB groups. C) LEfSe analysis of microbial taxa differentially abundant between HB and LB groups. D) Genus-level compositional differences between HB and LB groups.

**Figure S31.** Correlation between butyrate and microbial features. A) Correlation between butyrate intensity and the relative abundance of *Blautia* in the cecum. B) Correlation between butyrate intensity and the relative abundance of *Christensenellaceae_R-7_group* in the cecum. C) Correlation between butyrate intensity and the *Blautia/Christensenellaceae_R-7_group* (B/C) ratio. D) Receiver operating characteristic (ROC) curves for microbial biomarkers: *Blautia* (colored in blue), *Christensenellaceae_R-7_group* (colored in red), and B/C ratio (colored in yellow).

**Figure S32.** Genomic context of the L-cysteine associated loci on chromosome 2. A) Distribution of loci and genes associated with L-cysteine on chromosome 2. B) Chromatin states surrounding the candidate region associated with L-cysteine.

**Figure S33.** Cross-tissue correlation between gene expression and metabolites. A-C) Correlation with *MSRA* expression and butyrate intensity in A) heart, B) liver, and C) spleen tissues. D-F) Correlation with *PTER* expression and L-cysteine intensity in D) heart, E) liver, and F) spleen tissues.

**Figure S34.** Determination of half maximal inhibitory concentration for H_2_O_2_, ML385, IR-61, and L-cysteine.

**Figure S35.** Protein expression of Nrf2 pathway components across treatments. A) Nrf2. B) NQO1. C) HO-1. Values are expressed as the mean ± standard error. Data were analyzed by one-way ANOVA followed by Fisher’s LSD test. Different letters indicate significant differences (*P* < 0.05).

**
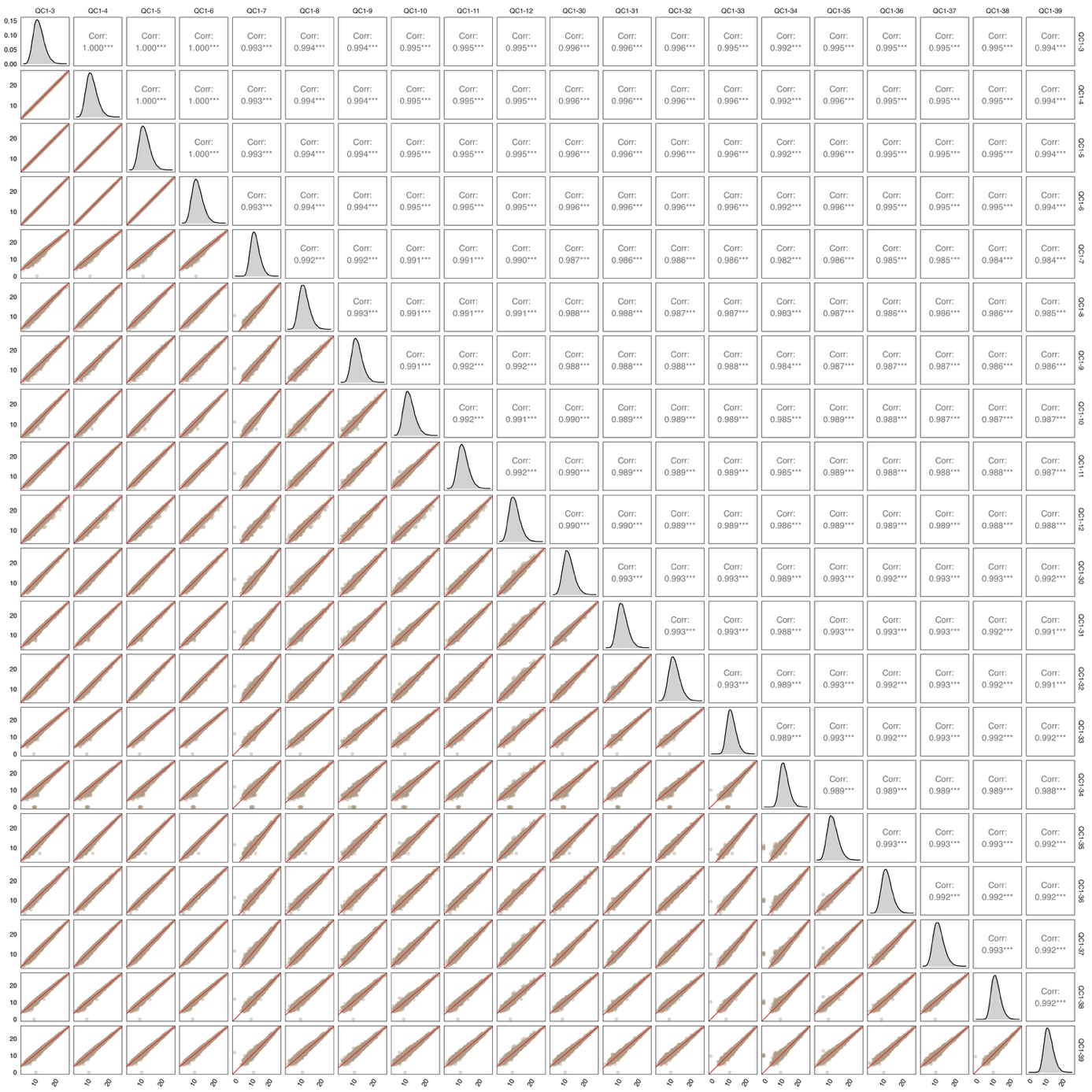
**

**Figure S36.** Correlation matrix illustrating the high reproducibility among 20 quality control samples.

**3.** **Supplementary Tables (in a separate Excel file)**

Table S1. Results of metabolite set enrichment analysis.

Table S2. The OR values of metabolic biomarkers identified by LASSO regression.

Table S3. Identified metabolite biomarkers and their chemical annotation.

Table S4. Compendium of human metabolic markers associated with aging, acute pathological processes, and cytokine responses.

Table S5. Feed ingredients for chicken during the experiments.

Table S6. The gradient program of ultra-performance liquid chromatography-tandem mass spectrometry system (comprised an ultra-performance liquid chromatography system and a high-resolution mass spectrometer).

Table S7. Quality statistics of the transcriptome sequencing data.

**4. Supplementary References**

1. J. R. Montenegro-Burke, C. Guijas, G. Siuzdak, “METLIN: A Tandem Mass Spectral Library of Standards,” *Methods Mol Biol* 2020, 2104, 149, https://doi.org/10.1007/978-1-0716-0239-3_9.

2. V. B. O'Donnell, E. A. Dennis, M. J. O. Wakelam, S. Subramaniam, “LIPID MAPS: Serving the next generation of lipid researchers with tools, resources, data, and training,” *Sci Signal* 2019, 12 (563), https://doi.org/10.1126/scisignal.aaw2964.

3. D. S. Wishart, A. Guo, E. Oler, et al., “HMDB 5.0: the Human Metabolome Database for 2022,” *Nucleic Acids Res* 2022, 50 (D1), D622, https://doi.org/10.1093/nar/gkab1062.

4. M. S. Tabatabaei, M. Ahmed, “Enzyme-Linked Immunosorbent Assay (ELISA),” *Methods Mol Biol* 2022, 2508, 115, https://doi.org/10.1007/978-1-0716-2376-3_10.

5. S. Chen, Y. Zhou, Y. Chen, J. Gu, “fastp: an ultra-fast all-in-one FASTQ preprocessor,” *Bioinformatics* 2018, 34 (17), i884, https://doi.org/10.1093/bioinformatics/bty560.

6. K. I. Kendig, S. Baheti, M. A. Bockol, et al., “Sentieon DNASeq Variant Calling Workflow Demonstrates Strong Computational Performance and Accuracy,” *Front Genet* 2019, 10, 736, https://doi.org/10.3389/fgene.2019.00736.

7. C. C. Chang, C. C. Chow, L. C. Tellier, et al., “Second-generation PLINK: rising to the challenge of larger and richer datasets,” *Gigascience* 2015, 4, 7, https://doi.org/10.1186/s13742-015-0047-8.

8. C. Zhang, S. S. Dong, J. Y. Xu, W. M. He, T. L. Yang, “PopLDdecay: a fast and effective tool for linkage disequilibrium decay analysis based on variant call format files,” *Bioinformatics* 2019, 35 (10), 1786, https://doi.org/10.1093/bioinformatics/bty875.

9. H. Shim, D. I. Chasman, J. D. Smith, et al., “A multivariate genome-wide association analysis of 10 LDL subfractions, and their response to statin treatment, in 1868 Caucasians,” *PLoS One* 2015, 10 (4), e0120758, https://doi.org/10.1371/journal.pone.0120758.

10. C. Gross, C. Bortoluzzi, D. de Ridder, et al., “Prioritizing sequence variants in conserved non-coding elements in the chicken genome using chCADD,” *PLoS Genet* 2020, 16 (9), e1009027, https://doi.org/10.1371/journal.pgen.1009027.

11. P. Cingolani, V. M. Patel, M. Coon, et al., “Using Drosophila melanogaster as a Model for Genotoxic Chemical Mutational Studies with a New Program, SnpSift,” *Front Genet* 2012, 3, 35, https://doi.org/10.3389/fgene.2012.00035.

12. P. Cingolani, “Variant Annotation and Functional Prediction: SnpEff,” *Methods Mol Biol* 2022, 2493, 289, https://doi.org/10.1007/978-1-0716-2293-3_19.

13. D. Kim, B. Langmead, S. L. Salzberg, “HISAT: a fast spliced aligner with low memory requirements,” *Nat Methods* 2015, 12 (4), 357, https://doi.org/10.1038/nmeth.3317.

14. S. Anders, P. T. Pyl, W. Huber, “HTSeq--a Python framework to work with high-throughput sequencing data,” *Bioinformatics* 2015, 31 (2), 166, https://doi.org/10.1093/bioinformatics/btu638.

15. Z. He, R. Liu, M. Wang, et al., “Combined effect of microbially derived cecal SCFA and host genetics on feed efficiency in broiler chickens,” *Microbiome* 2023, 11 (1), 198, https://doi.org/10.1186/s40168-023-01627-6.

16. C. Cheng, F. Xu, X. F. Pan, et al., “Genetic mapping of serum metabolome to chronic diseases among Han Chinese,” *Cell Genom* 2025, 5 (2), 100743, https://doi.org/10.1016/j.xgen.2024.100743.

17. F. Wang, A. J. Tessier, L. Liang, et al., “Plasma metabolomic profiles associated with mortality and longevity in a prospective analysis of 13,512 individuals,” *Nat Commun* 2023, 14 (1), 5744, https://doi.org/10.1038/s41467-023-41515-z.

18. Y. Zhang, C. Quick, K. Yu, et al., “PTWAS: investigating tissue-relevant causal molecular mechanisms of complex traits using probabilistic TWAS analysis,” *Genome Biol* 2020, 21 (1), 232, https://doi.org/10.1186/s13059-020-02026-y.

19. S. Park, S. Kim, B. Kim, et al., “Multivariate genomic analysis of 5 million people elucidates the genetic architecture of shared components of the metabolic syndrome,” *Nat Genet* 2024, 56 (11), 2380, https://doi.org/10.1038/s41588-024-01933-1.
